# The Genetic Landscape of Complex Childhood‐Onset Hyperkinetic Movement Disorders

**DOI:** 10.1002/mds.29182

**Published:** 2022-08-25

**Authors:** Belén Pérez‐Dueñas, Kathleen Gorman, Anna Marcé‐Grau, Juan D. Ortigoza‐Escobar, Alfons Macaya, Federica R. Danti, Katy Barwick, Apostolos Papandreou, Joanne Ng, Esther Meyer, Shekeeb S. Mohammad, Martin Smith, Francesco Muntoni, Pinki Munot, Johanna Uusimaa, Päivi Vieira, Eammon Sheridan, Renzo Guerrini, Jan Cobben, Sanem Yilmaz, Elisa De Grandis, Russell C. Dale, Roser Pons, Kathryn J. Peall, Vincenzo Leuzzi, Manju A. Kurian

**Affiliations:** ^1^ Department of Pediatric Neurology Vall d'Hebron Hospital Universitary and Vall d'Hebrón Research Institute (VHIR). Barcelona Spain; ^2^ Department of Pediatrics, Obstetrics, Gynecology, Preventative Medicine and Public Health Universitat Autònoma de Barcelona Barcelona Spain; ^3^ Center for Biomedical Network Research on Rare Diseases (CIBERER) CB06/07/0063 Barcelona Spain; ^4^ Developmental Neurosciences Programme Great Ormond Street–Institute of Child Health, University College London London United Kingdom; ^5^ Dubowitz neuromuscular Center Great Ormond Street Hospital for Children London United Kingdom; ^6^ Department of Pediatric Neurology Sant Joan de Déu Hospital Barcelona Spain; ^7^ Unit of Child Neurology and Psychiatry, Department of Human Neuroscience Sapienza University of Rome Rome Italy; ^8^ Gene Transfer Technology Group Institute for Women's Health, University College London London United Kingdom; ^9^ Kids Neuroscience Centre and Brain and Mind Centre, Faculty of Medicine and Health University of Sydney Westmead New South Wales Australia; ^10^ Department of Pediatric Neurology John Radcliffe Hospital Oxford United Kingdom; ^11^ PEDEGO Research Unit, Department of Children and Adolescents, Medical Research Center Oulu Oulu University Hospital, University of Oulu Oulu Finland; ^12^ School of Medicine St James's University Hospital, University of Leeds Leeds United Kingdom; ^13^ Pediatric Neurology, Neurogenetics and Neurobiology Unit and Laboratories, Neuroscience Department A. Meyer Children's Hospital, University of Florence Florence Italy; ^14^ North West Thames Regional Genetic Service Northwick Park Hospital London United Kingdom; ^15^ Department of Pediatrics, Division of Child Neurology Ege University Medical Faculty İzmir Turkey; ^16^ Child Neuropsychiatry Unit, Istituto Giannina Gaslini, Department of Neurosciences, Rehabilitation, Ophthalmology, Genetics and Maternal and Children's Sciences University of Genoa Genoa Italy; ^17^ Institute for Neuroscience and Muscle Research Children's Hospital at Westmead, University of Sydney Sydney New South Wales Australia; ^18^ First Department of Pediatrics Agia Sofia Children's Hospital, National and Kapodistrian University of Athens Athens Greece; ^19^ Neuroscience and Mental Health Research Institute Institute of Psychological Medicine and Clinical Neurosciences, School of Medicine, Cardiff University Cardiff United Kingdom

**Keywords:** dystonia, chorea, myoclonus, infantile parkinsonism, hyperkinetic movement disorders

## Abstract

**Background and Objective:**

The objective of this study was to better delineate the genetic landscape and key clinical characteristics of complex, early‐onset, monogenic hyperkinetic movement disorders.

**Methods:**

Patients were recruited from 14 international centers. Participating clinicians completed standardized proformas capturing demographic, clinical, and genetic data. Two pediatric movement disorder experts reviewed available video footage, classifying hyperkinetic movements according to published criteria.

**Results:**

One hundred forty patients with pathogenic variants in 17 different genes (*ADCY5*, *ATP1A3*, *DDC*, *DHPR*, *FOXG1*, *GCH1*, *GNAO1*, *KMT2B*, *MICU1*, *NKX2*.*1*, *PDE10A*, *PTPS*, *SGCE*, *SLC2A1*, *SLC6A3*, *SPR*, and *TH*) were identified. In the majority, hyperkinetic movements were generalized (77%), with most patients (69%) manifesting combined motor semiologies. Parkinsonism‐dystonia was characteristic of primary neurotransmitter disorders (*DDC*, *DHPR*, *PTPS*, *SLC6A3*, *SPR*, *TH*); chorea predominated in *ADCY5*‐, *ATP1A3*‐, *FOXG1*‐, *NKX2*.*1*‐, *SLC2A1*‐, *GNAO1*‐, and *PDE10A*‐related disorders; and stereotypies were a prominent feature in *FOXG1*‐ and *GNAO1*‐related disease. Those with generalized hyperkinetic movements had an earlier disease onset than those with focal/segmental distribution (2.5 ± 0.3 vs. 4.7 ± 0.7 years; *P* = 0.007). Patients with developmental delay also presented with hyperkinetic movements earlier than those with normal neurodevelopment (1.5 ± 2.9 vs. 4.7 ± 3.8 years; *P* < 0.001). Effective disease‐specific therapies included dopaminergic agents for neurotransmitters disorders, ketogenic diet for glucose transporter deficiency, and deep brain stimulation for *SGCE*‐, *KMT2B*‐, and *GNAO1*‐related hyperkinesia.

**Conclusions:**

This study highlights the complex phenotypes observed in children with genetic hyperkinetic movement disorders that can lead to diagnostic difficulty. We provide a comprehensive analysis of motor semiology to guide physicians in the genetic investigation of these patients, to facilitate early diagnosis, precision medicine treatments, and genetic counseling. © 2022 The Authors. *Movement Disorders* published by Wiley Periodicals LLC on behalf of International Parkinson and Movement Disorder Society

Hyperkinetic movement disorders (HMDs) encompass a broad spectrum of complex diseases, frequently associated with motor disability. Hyperkinesia is characterized by involuntary, excessive movements or abnormal muscle activity during active movement.^1^ A number of different hyperkinetic phenotypes are described, including dystonia, chorea, athetosis, myoclonus, tremor, tics, and stereotypies. Accurate terminology is essential to facilitate diagnosis, therapeutic choices, and communication between professionals.

Despite recent major advances in molecular genetics, children presenting with suspected genetic HMDs often remain a diagnostic challenge. Many of them have a combined hyperkinetic motor semiology that can be difficult to accurately categorize, even for the experienced movement disorder specialist. Associated comorbidities, such as developmental delay and other neurological or systemic features, frequently coexist, causing complex HMD phenotypes that are difficult to recognize.

The differential diagnosis is often broad, given the phenotypic overlap with hypoxic‐ischemic encephalopathy, autoimmune diseases, infection, stroke, and metabolic disorders. For many complex genetic HMDs, there are currently no disease biomarkers or radiological clues to aid the diagnostic odyssey. In addition, with growing genetic heterogeneity and phenotypic pleiotropy, it has become increasingly difficult to predict genotype from clinical phenotype. Nonetheless, in this genomic era, the importance of accurate and detailed endophenotyping to assist correct interpretation of genomic variants cannot be underestimated.

In this study, we aim to better characterize the genetic landscape of complex childhood HMDs, with a focus on disorders *without* either blood and urine biomarkers or *extensive* structural abnormalities of the basal ganglia. Through this work, we provide a comprehensive analysis of motor semiology, as well as detailed information about associated key neurological and systemic features, to better guide and interpret genetic investigations, thereby facilitating accurate diagnosis, prognostication, and future genetic counseling.

## Patients and Methods

### Study Design

Nineteen international tertiary pediatric movement disorder centers were invited to participate in the study, of which 14 contributed patients (Supplementary Data in Appendix S1).

### Ethical Approval and Consent

Appropriate ethical approvals were in place at all participating centers (Supplementary Data in Appendix S1). Written informed consent was obtained for publication of video footage.

### Study Inclusion Criteria

Individual patient criteria included: (1) patient's age at motor symptom onset <18 years; (2) video footage of entire body (head, neck, trunk, and limbs) available for expert clinical review; and (3) confirmation of genetic etiology as defined by American College of Medical Genetics and Genomics guidelines.^2^For each genetic disorder, the reported literature confirms that (1) HMD is the main motor feature with either one (pure HMD phenotype) or more (combined HMD phenotype) of the following movement phenotypes: dystonia, chorea, athetosis, myoclonus, or tremor; (2) HMD is not purely paroxysmal; (3) HMD is commonly associated with other neurological, neuropsychiatric, or systemic comorbidities; (4) there are no highly predictive blood or urine biomarkers for the gene in question; and (5) there is no major basal ganglia structural abnormality (or only subtle findings) on neuroimaging for the gene in question.

### Data Acquisition

Video footage was obtained during clinic appointments, as part of routine clinical care, and was considered to be representative of the HMD by the treating clinician. Only videos showing footage of the entire body were selected for review. Videos were reviewed by two child neurologists with expertise in movement disorders (B.P.‐D. and M.A.K.). The observed HMD semiology was classified according to published consensus guidelines.^1^ The body distribution was recorded as focal, segmental, or generalized. Other coexisting non‐HMDs, such as parkinsonism and ataxia, were also recorded.

Clinical data were collected through an anonymized standardized proforma to record age at assessment, sex, ethnicity, consanguinity, other affected family members, causative gene and mutation, mode of inheritance, and gene‐related phenotype. We also gathered data regarding age at HMD onset, disease evolution, paroxysmal or fluctuating HMD symptoms, triggers, other neurological or systemic features, and response to treatment. The functional impact of HMD on daily living activities (DLAs) was scored (Supplementary Data in Appendix S1).

### Genetic Analysis

All patients had been previously diagnosed with pathogenic or likely pathogenic variants in genes reported to be associated with complex HMD genes, using standard American College of Medical Genetics and Genomics guidelines.^2^ Genetic diagnoses were made by (1) array comparative genomic hybridization (aCGH) (n = 14), (2) targeted diagnostic or research Sanger sequencing of single genes (n = 85), (3) diagnostic multiple gene panels (n = 18), (4) research whole‐exome sequencing (n = 19), and (5) research whole‐genome sequencing (n = 4) (Table S1 in Appendix S1).

### Statistical Analysis

The SPSS v.24.0 (IBM Corp., Armonk, N.Y., USA) statistical package was used to calculate means, standard deviations, and ranges. Based on small sample size, the Mann–Whitney *U* test was chosen to compare continuous variables (ie, age at disease onset) between different phenotypic groups. Differences were reported as significant when *P* < 0.05.

## Results

Retrospective data analysis was undertaken on 140 patients with complex genetic HMD (Table S1 in Appendix S1). The mean age of HMD onset was 2.8 (range 0–17) years. Mean patient age at last clinical assessment was 13.3 (range 0.5–68) years. Male/female ratio was relatively equal (66:74). A total of 96/140 (68.6%) patients have been previously reported in the literature.

### Molecular Genetic Findings

Seventeen different genetic etiologies were identified (Table S1 in Appendix S1). Forty‐two patients had biallelic variants in eight genes, including those causing recessive neurotransmitter defects (*DDC*, *DHPR*, *PTPS*, *SLC6A3*, *SPR*, and *TH*) and *PDE10A*‐ and *MICU1‐*related disease. The remaining 98 patients had autosomal dominant variants in nine genes (*ADCY5*, *ATP1A3*, *FOXG1*, *GCH1*, *GNAO1*, *KMT2B*, *NKX2*.*1*, *SGCE*, *SLC2A1*) that were either inherited or had occurred de novo. Within the cohort, all parents harboring the same *ATP1A3*, *NKX2*.*1*, or *SLC2A1* variant as their affected child were also symptomatic. In contrast, incomplete penetrance was observed for *ADCY5‐*, *KMT2B‐*, *GCH1‐*, and *SGCE‐*related HMDs. Although the majority (126/140) of patients had intragenic variants, microdeletions encompassing the causative gene were identified on aCGH in 14 patients with *FOXG1‐*, *KMT2B‐*, *NKX2*.*1‐*, and *SGCE*‐related HMDs.

**FIG 1:**
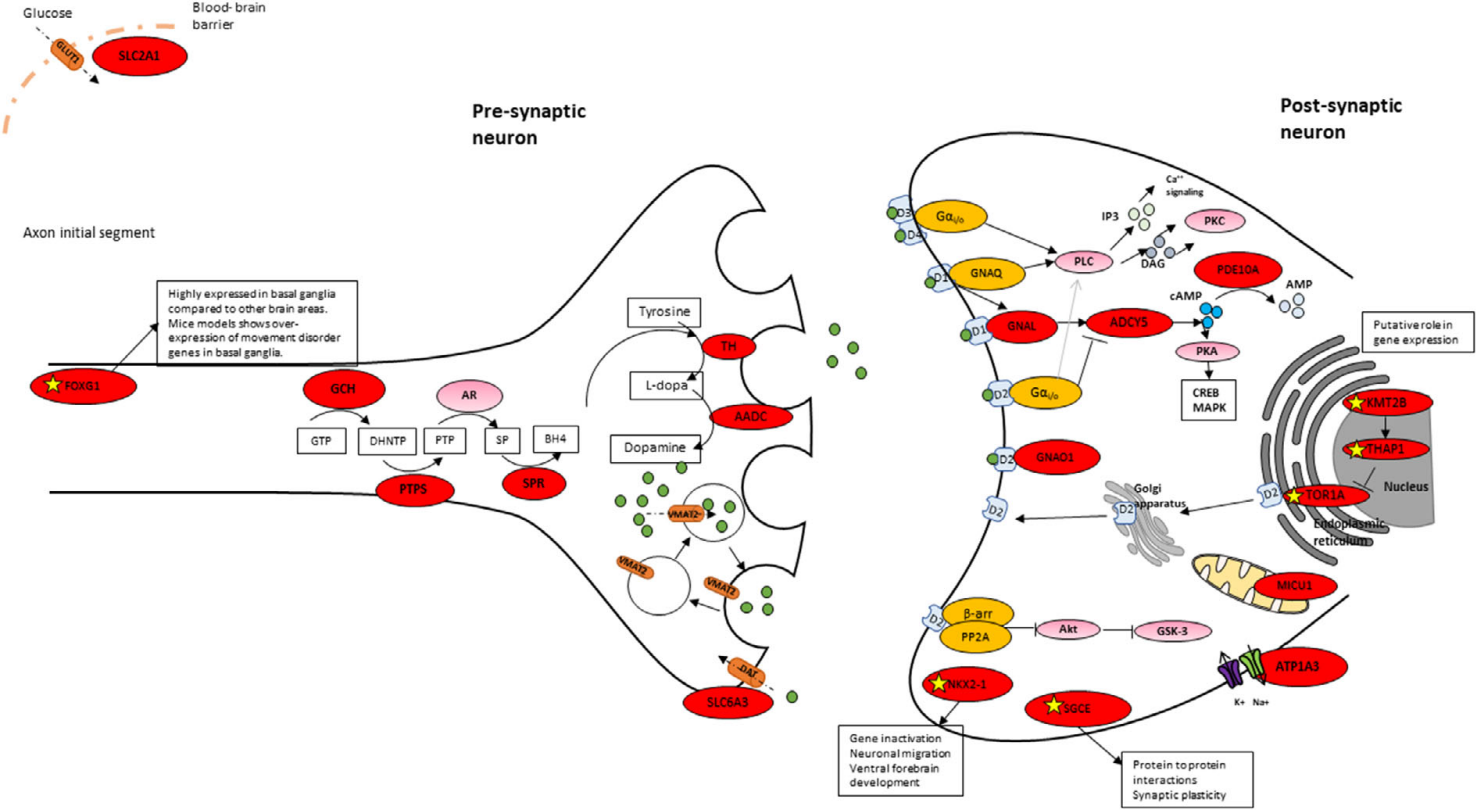
Proposed disease pathways involved in genetic hyperkinetic movement disorders (HMDs) of childhood onset: schematic representation of neuronal synapse and key proteins postulated to be involved in motor control. Yellow star: mechanism in dystonia not fully elucidated for this gene, postulated mechanism, or function not known; red circle: gene reported in association with complex genetic HMDs in this article; pink circle: gene not reported in this article; green circle: dopamine; gray circles: DAG, cAMP, and AMP as indicated in figure. Akt, protein kinase B; AMP, adenosine 3′,5′‐monophosphate; AR, aldose reductase; β‐arr, β‐arrestin; BH4, tetrahydrobiopterin; cAMP, cyclic AMP; CREB, cAMP‐response element binding; DAG, diacylglycerol; DAT, dopamine transporter; DNHTP, 7,8‐dihydroneopterin triphosphate; GLUT1, glucose transporter 1; GNAQ, guanine nucleotide‐binding protein G(q); GSK‐3, glycogen synthase kinase 3; GTP, guanosine‐5′‐triphosphate; IP_3_, inositol 1,4,5‐triphosphate; MAPK, mitogen‐activated protein kinase; PKA, protein kinase A; PKC, protein kinase C; PLC, phospholipase C; PP2A, protein phosphatase 2; PTP, 6‐pyruvoyl tetrahydropterin; SP, sepiapterin reductase; *VMAT2*, vesicular monoamine transporter 2. [Color figure can be viewed at wileyonlinelibrary.com]

Variants in a broad range of genes were identified, with key neuronal roles, including regulation of transcription, neuronal differentiation, synaptic transmission, postsynaptic G protein–cAMP signaling, ion flux, dopamine homeostasis, glucose transport, and maintenance of the dystrophin–glycoprotein complex (Fig. 1).

### HMD Classification and Body Distribution

**FIG 2:**
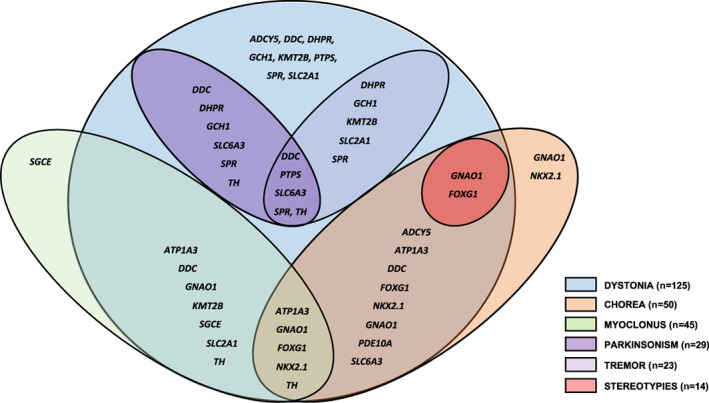
Venn diagram that represents the different combinations of movement disorders that were linked to each genetic defect in our series. [Color figure can be viewed at wileyonlinelibrary.com]

**FIG 3:**
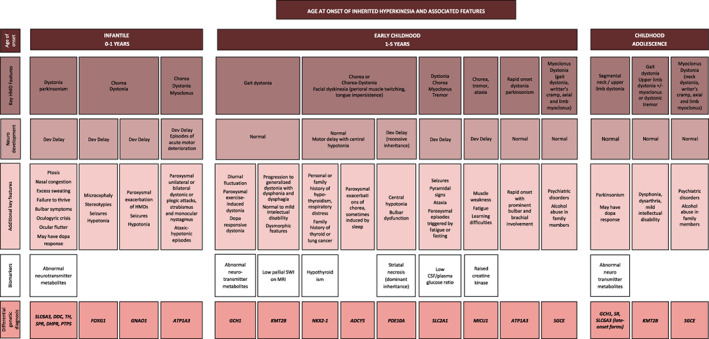
Flow diagram with the age at onset, key hyperkinetic motor semiology, and key associated neurological and systemic features identified in our series. We also include biochemical and radiological biomarkers that are valuable for the differential genetic diagnosis. CSF, cerebrospinal fluid; Dev, developmental; HMD, hyperkinetic movement disorder; MRI, magnetic resonance imaging; SWI, susceptibility weighted imaging. [Color figure can be viewed at wileyonlinelibrary.com]

Figures 2 and 3 illustrate two diagrams with the different combinations of movement disorders linked to each genetic defect (Fig. 2) and the age at onset, key hyperkinetic motor semiology, and key associated neurological and systemic features identified in our series (Fig. 3). The majority of patients (96/140, 68.6%) had two or more coexisting HMDs (two HMDs, n = 68; three HMDs, n = 27; four HMDs, n = 1) (Video S1). Dystonia was the most frequently reported HMD (n = 125), followed by chorea‐ballism (n = 50), myoclonus (n = 45), tremor (n = 23), and stereotypies (n = 18) (Tables 1 and 2). Tics were identified in one patient. Patients with HMDs also showed other coexisting non‐HMDs, such as parkinsonism (n = 29) and ataxia (n = 9). Pure HMD phenotypes were observed in 35 patients (pure dystonia, n = 25; pure chorea, n = 5; pure myoclonus, n = 5) (Tables 1 and 2).

**TABLE 1:** Age at onset, motor semiology, body distribution, and effective treatments for complex genetic HMDs identified in our cohort

Gene (no. of patients)	Average age at onset of HMDs (range), y	Average age at last assessment (range), y	No. of patients with HMDs according to consensus guidelines	No. of patients with facial HMDs	Distribution of the HMDs and coexistence with ataxia/parkinsonism	Effective treatment for HMDs
*ADCY5* (n = 4)	1.3 (0.5–2.5)	6.7 (3–11)	Dystonia (4) Chorea (3)	Facial dyskinesia (perioral) (3) Tongue impersistence (3)	Generalized chorea (F, T, L) (3) Focal: CC (1)	Yes – Transient and partial improvement with valproate (1), acetazolamide (1), carbamazepine (1)
*ATP1A3* (n = 6)	0.3 (0.08–4)	12 (8–30)	Dystonia (6) Chorea (5) Myoclonus (3) Ballismus (1)	Facial dyskinesias (4) OM dystonia (1)	Generalized (F, T, L) in all Ataxia (3) Parkinsonism (1)	Yes – Flunarizine (4), topiramate (1), and diazepam (1) decreased paroxysmal episodes and improved development in one patient
*FOXG1* (n = 17)	0.6 (0–1)	11 (3–25)	Chorea (16) Dystonia (16) Stereotypies (14) Myoclonus (2)	Facial dyskinesia (grimacing/lip pursing) (12) Jaw dystonia and tongue protrusion (12)	Generalized (F, T, L) in all Parkinsonism (1)	Yes – Levodopa reduced hyperkinesia and increased dexterity in four patients
*GNAO1* (n = 7)	2.3 (0.3–8.9)	5.4 (0.6–14)	Chorea (7) Dystonia (5) Ballismus (3) Stereotypies (3) Myoclonus (1)	Perioral dyskinesia (5) Eye blinking (1)	Generalized (F, T, L) in all	Yes – Tetrabenazine (2) and DBS (1) improved movement disorder
*KMT2B* (n = 17)	8.1 (1–43)	20 (8–60)	Dystonia (17) Tremor (3) Myoclonus (2)	Orolingual dystonia (2)	Generalized (F, T, L) in all	Yes – DBS inserted in nine patients with considerable benefit
*MICU1* (n = 2)	2.5 (2–3)	14–20	Chorea (2) Tremor (2)	Facial dyskinesias (1)	Generalized (F, T, L) in both Sensory/paroxysmal ataxia (2)	None mentioned
*NKX2*.*1* (n = 8)	3 (1–7)	12 (4–18)	Chorea (8) Dystonia (4) Myoclonus (2)	Tongue impersistence (4) Facial dyskinesia (2) OM dystonia (1)	Generalized (F, T, L) in all Ataxia (2)	Yes – Levodopa had a transient and partial improvement in chorea and gait (1)
*PDE10A* (n = 2)	0.3	7.5 (7–8)	Chorea (2) Dystonia (2)	OM dystonia (2)	Generalized (F, T, L) in both	No
*SGCE* (n = 30)	4 (1.3–11)	25 (2–68)	Myoclonus‐dystonia (25) Myoclonus (5)	Excessive blinking (7) Chin jerks (6)	Generalized (9) Focal/segmental (21) Areas involved: CC (22), ULs (26), axial (24), LLs (12)	Yes – DBS significantly improved dystonia and myoclonus (3)
*SLC2A1* (n = 4)	2.9 (1.5–5)	7.7 (4–14)	Dystonia (3) Chorea (1) Myoclonus (1) Tremor (1)	Facial dyskinesia (2)	Generalized (2) Ataxia (2) Focal: ULs (1), CC (1)	Yes – Ketogenic diet improved motor performance and paroxysmal episodes (4)
**Neurotransmitters defects**						
*DDC* (n = 9)	0.27 (0–0.75)	9.2 (0.6–32)	Dystonia (9) Myoclonus (2) Chorea/Ballismus (2) Tremor (1)	Facial dyskinesias (5) OM dystonia (3) Eye rolling (2)	Generalized (F, T, L) (7) Segmental: ULs (1) Parkinsonism (4)	Yes – Dopamine agonists (9) and MAOIs (3) improved oculogyric crisis and motor function in all patients
*DHPR* (n = 4)	0.8 (0–2)	3 (0.5–10)	Dystonia (4) Kinetic tremor (1)	Facial dyskinesia of jaw and tongue (2)	Generalized (F, T, L) in all patients Parkinsonism (2)	Yes – Levodopa, 5‐HT, vitamin B_6_ MAOI – partial relief of motor symptoms in all
*GCH* (n = 6)	6 (2–15)	12.8 (4–41)	Dystonia (6) Dystonic tremor (1)	Facial dyskinesia (1)	Generalized (F, T, L) (5) Focal: LLs (3) Parkinsonism (1)	Yes – Levodopa led to significant and lasting improvement in all
*PTPS* (n = 4)	4.2 (0.1–16)	9.8 (0.6–32)	Dystonia (4) Tremor (2)	OM dystonia (3) Down gaze deviation (1)	Generalized (F, T, L) (3) Segmental (UL, CC) (1) Parkinsonism (2)	Yes – Tetrahydrobiopterin (4), levodopa (3), 5‐HT (2), MAOIs (2) improved motor function
*SLC6A3* (n = 10)	4.2 (0.04–11)	10 (2–28)	Dystonia (10) Rest tremor (7) Chorea (3) Ballismus (1)	Ocular flutter (4) Eyelid myoclonus (3) Facial grimacing (4) OM dystonia (3)	Generalized (F, T, L) (8) Focal (CC) (2) Parkinsonism (8)	Yes – Ropinirole (3) and pramipexole (1) caused mild and transient improvement in motor symptoms
*SPR* (n = 4)	10.3 (0.5–15)	1.5 (0–5)	Dystonia (4) Tremor (2)	Facial and tongue dyskinesia (2)	Generalized (F, T, L) (3) Focal dystonia (CC and OGCs) (1) Parkinsonism (2)	Yes – Levodopa stopped oculogyric crisis and led to progressive improvement in motor development in all patients
*TH* (n = 7)	0.4 (0.2–1)	3.7 (0.5–13)	Dystonia (7) Myoclonus (3) Tremor (3) Chorea (2) Ballismus (1)	Facial and tongue dyskinesias (3)	Generalized (F, T, L) in all Parkinsonism (7)	Yes – Levodopa stopped oculogyric crisis and led to progressive improvement in all areas of neurodevelopment in all patients

Age at onset and age at last assessment are reported in years. Distribution of the movement disorder: face (F), trunk (T), limbs (L), upper limbs (UL), lower limbs (LL), and craniocervical (CC).

HMD, hyperkinetic movement disorder; OM, oromandibular; DBS, deep brain stimulation; MAOI, monoamine oxidase inhibitor; Oculogyric crisis (OGCs)5‐hydroxytryptophan (5‐HT).

**TABLE 2:** Prevalence of different movement phenotypes in complex genetic hyperkinetic movement disorders

Genes	Chorea	Dystonia	Myoclonus	Tremor	Parkinsonism	Ataxia	Stereotypies
*ADCY5*							
*ATP1A3*							
*FOXG1*							
*GNAO1*							
*KMT2B*							
*MICU1*							
*NKX2*.*1*							
*PDE10A*							
*SGCE*							
*SLC2A1*							
Neurotransmitter defects							
*DDC*							
*DHPR*							
*GCH*							
*PTPS*							
*SLC6A3*							
*SPR*							
*TH*							

0–24%	25–49%	50–74%	75–100%

Color shading indicates the percentage of patients with this movement disorder.

The majority of patients (108/140, 77%) had a generalized HMD (Table 1, Fig. 2). Focal or segmental HMD was observed in only 32/140 (23%) patients with *SGCE*, *SLC2A1*, *ADCY5*, and atypical, later‐onset neurotransmitters disorders (*GCH1*, *SLC6A3*, *DDC*, *PTPS*, and *SPR*). Patients with focal semiology had significantly later onset of HMD than those with generalized symptoms (4.7 ± 0.7 vs. 2.5 ± 0.3 years, respectively; *P* = 0.001).

#### Abnormal Eye Movements

Oculogyric crises were commonly seen in all neurotransmitter defects except autosomal dominant Segawa disease. Eyelid myoclonus and ocular flutter were evident in DTDS(Dopamine Transporter Deficiency Syndrome). Ptosis (TH and AADC (Aromatic L‐amino acid decarboxylase) deficiency) and oculomotor dyspraxia (TH deficiency) were also observed. Within the wider cohort, strabismus (*FOXG1* and *ATP1A3*) and nystagmus (*MICU1*) were also reported. Tonic upgaze associated with severe retrocollis was observed in one patient with *KMT2B*‐dystonia.

#### Hyperkinetic Facial Movements

Facial hyperkinesia was observed in 80 patients, affecting the upper (n = 31) and/or lower face (n = 66). Forehead wrinkling, excessive blinking, facial grimacing, perioral muscle twitching, lip pursing, jaw opening or clenching, and tongue thrusting, dyskinesia, or impersistence were observed. Facial hyperkinesia was more frequent in children with *FOXG1‐*, *ADCY5‐*, *NKX2*.*1‐*, and *GNAO1‐*related HMD, as well as AADC deficiency and DTDS.

#### Dystonia

Pure dystonia was observed in 25 patients (Fig. 2). For the remainder, dystonia was always combined with other HMDs, namely, chorea (n = 40), myoclonus (n = 40), tremor (n = 20), and stereotypies (n = 17) (Fig. 2, Table 1). Overall, within the cohort, a number of distinct dystonia phenotypes were evident, including pure dystonia, chorea‐dystonia, myoclonus‐dystonia, and dystonia‐parkinsonism (Video S2).

Dystonia of the lower limbs was observed in 58 ambulant and 27 nonambulant patients. In the nonambulant group, bilateral dystonic postures included fixed/variable leg flexion/extension, striatal toe, toe clawing, and clenched feet. In the ambulant group, patients had gait dystonia with lower‐limb posturing, toe‐walking, striatal toe, toe‐clawing, and gait disturbance. All patients with *KMT2B*‐HMD had lower‐limb symptoms at disease onset, leading to a purely dystonic gait. Lower‐limb dystonia was also observed in 21/61 patients with neurotransmitter defects and in 10/30 SGCE patients, the latter associated with myoclonic jerks in three patients. In the remaining patients, gait dystonia was associated with chorea (*ATP1A3*, *GNAO1*, and *NKX2*.*1*) and ataxia (*ATP1A3*, *NKX2*.*1*, and *SLC2A1*).

Upper‐limb dystonia was observed in 98/140 patients, with fixed/variable dystonic posturing of the arms, hand fisting, superimposed coarse dystonic tremor, and action‐induced writer's cramp.

Cervical dystonia was evident in 50 patients (neurotransmitter defects, *SGCE*, *KMT2B*, *FOXG1*, *ATP1A3*, *ADCY5*, and *SLC2A1*).

#### Chorea

Generalized chorea was observed in 50 patients (Fig. 2). Pure chorea was seen in only five patients with *GNAO1*‐ and *NKX2*.*1*‐related HMD, while in the remainder, chorea was associated with other HMDs (dystonia, n = 36; stereotypies, n = 16; myoclonus, n = 9; ballismus, n = 4; tremor, n = 2; tics, n = 1). Chorea was the predominant HMD in *ADCY5‐* (n = 4), *ATP1A3‐* (n = 5), *FOXG1‐* (n = 16), *GNAO1‐* (n = 7), *MICU1‐* (n = 2), *NKX2*.*1‐* (n = 8), and *PDE10A‐*related (n = 2) disease. Chorea was also a prominent HMD in seven children with neurotransmitter defects (*DDC*, *TH*, *SLC6A3*), commonly in tandem with dystonia (n = 5), ballismus (n = 2), myoclonus (n = 1), coarse tremor (n = 3), and stereotypies (n = 1) (Video S3).

#### Myoclonus

Myoclonus was observed in 45 patients (Fig. 2). Pure myoclonus was observed in only five patients with *SGCE*‐related HMD, while in the other cases, myoclonus coexisted with dystonia (n = 40), chorea (n = 9), and tremor (n = 1) (Video S4).

#### Tremor

Tremor was observed in 23 patients, most commonly in those with neurotransmitter diseases, but also in *KMT2B‐*, *MICU1‐*, and *SLC2A1‐*related diseases (Fig. 2). Tremor was observed in combination with dystonia (n = 20), parkinsonism (n = 13), ataxia (n = 3), chorea (n = 2), and myoclonus (n = 2). In patients with neurotransmitters defects, a coarse generalized resting tremor was observed in nine infants with parkinsonism‐dystonia (Video S5). Focal neck and upper limb tremor were observed in seven patients with neurotransmitter defects (*DHPR*, *GCH1*, *PTPS*, *SLC6A3*, and *SPR*). Prominent head tremor was also identified in one child with Glut1 deficiency and in a patient with *KMT2B*‐dystonia with retrocollis.

#### Stereotypies

Fourteen patients with *FOXG1*‐related HMDs displayed complex motor stereotypies of the upper limbs (repetitive mouthing of hands/objects, midline hand wringing, grasping of clothes), lower limbs (pedalling/pulling), body rocking, bruxism, and nail biting. Three children with *GNAO1*‐related HMDs also showed self‐injurious stereotypic behavior (lip and nail biting, hair pulling) and repetitive nonpurposeful distal finger movements.

### Other Neurological and Systemic Features

Developmental delay was evident in 69/140 (49%) patients, being severe in 36 patients with neurotransmitter defects (*SLC6A3*, *TH*, *DHPR*, *DDC*) and in *GNAO1*‐, *FOXG1*‐, and *ATP1A3*‐related HMDs. Patients with developmental delay had an earlier age of HMD onset than those with normal neurodevelopment (1.5 ± 2.9 vs. 4.7 ± 3.8; *P* < 0.001). Epileptic seizures were reported in 32/140 (23%) patients and more commonly in those with *FOXG1*, *SLC2A1*, *GNAO1*, *ATP1A3*, and *DHPR* variants. Microcephaly was identified in 23/140 (16%) patients with *FOXG1*, *GNAO1*, *SLC2A1*, *SGCE*, and *MICU1* variants and some neurotransmitters disorders (*TH*, *PTPS*, *DDC*, *SPR*). Other associated neurological features included psychiatric problems (49/140 patients with anxiety, mood disorders, emotional lability, aggressive behavior, attention deficit hyperactivity disorder, autism spectrum disorder, obsessive–compulsive disorder, panic disorder, specific phobia), hypotonia (37/140), pyramidal signs (17/140), as well as systemic features (46/140 patients, ie, failure to thrive, short stature, dysmorphic features, autonomic dysfunction, gastrointestinal dysfunction, hypothyroidism, respiratory distress) (Table S2 in Appendix S1).

### Functional Impairment of HMDs and Impact on DLAs

Ninety‐five of 124 patients (77%) had gait impairment. Forty‐four were either nonambulant or able to walk only supported by another individual or assistive device. Fifty‐one patients were able to walk independently, but their gait was slow and/or frequently associated with balance difficulties and falling. Patients with *GNAO1*, *DDC*, *PDE10A*, *SLC6A3*, and *FOXG1* mutations had the highest average scores for gait impairment (2.9, 2.5, 2.5, 2.3, and 2.3, respectively), followed by those with *KMT2B* and *ATP1A3* (1.5), *TH* and *PTPS* (1.3), *GCH1*, *MICU1*, and *NKX2*.*1* (1), *SLC2A1* (0.6), and *ADCY5*, *DHPR*, and *SPR* (0.5) variants. Patients with *SGCE* mutations had the lowest average gait impairment score (0.36).

Speech impairment was recorded in 73/112 (65%) patients older than 3 years. The most affected groups (>75% of the cases) were those with *ATP1A3*‐, *FOXG1*‐, *GNAO1*‐, *KMT2B*‐, and *PDE10A*‐related HMDs and all patients with neurotransmitter defects with the exception of GCH1 deficiency (Table S3 in Appendix S1). Speech difficulties included dysarthria (all HMD genes), language delay (infantile‐onset *DDC*‐, *ATP1A3*‐, *FOXG1*‐, *GNAO1*‐, *PDE10A*‐, *SLC6A3*‐, and *TH*‐related HMDs), bradylalia (neurotransmitters defects), and dysphonia (*KMT2B*).

Patients with genetic HMDs also had difficulties with eating/swallowing (n = 54), as well as DLAs requiring fine motor control (n = 67) (Table S3 in Appendix S1).

### Paroxysmal Fluctuation

Oculogyric crises, triggered by hunger, illness, and fatigue, were recorded in 25/46 (54%) children with *DDC*‐, *DHPR*‐, *PTPS*‐, *SLC6A3*‐, *SPR*‐, and *TH*‐related HMDs. These children also had periodic dystonic attacks that were often associated with orolingual dyskinesia. Diurnal fluctuation of motor symptoms (associated with sleep benefit) was also described in this group. Gait difficulties, poor balance, and frequent falls triggered by fatigue and prolonged exercise (with partial relief of symptoms after sleep) were seen in five of eight patients with *GCH1* defects (Table S4 in Appendix S1).

In *ATP1A3* patients, paroxysmal paralysis, dystonia, ataxia, and dysphagia were commonly reported. Identified triggers were extremes of temperature, bathing, emotional stress, or fatigue. Paroxysmal exacerbation of baseline choreoathetosis was reported in two children with *ADCY5* variants, triggered by drowsiness and sleep in one. Episodes of acute ataxia, quadriplegia, and dystonic posturing were described in all four children with Glut1 deficiency after exercise, fatigue, prolonged fasting, and febrile illness. Patients with *GNAO1* mutations showed exacerbation of hyperkinetic movements with intercurrent infection, heightened emotion, and purposeful movements. Periods of severe exacerbation were characterized by relentless hyperkinesia, often necessitating admission to the intensive care unit. Dystonic crises were also reported in patients with *KMT2B*‐related disease (n = 2) and *SLC6A3*‐related disorders (n = 4), most commonly triggered by intercurrent illness.

### Treatment and Disease Course

Thirty‐four of 45 patients with neurotransmitters defects showed varying degrees of clinical improvement with dopaminergic agents (levodopa [l‐dopa], dopamine agonists, monoamine oxidase inhibitors). Of the remaining 11 patients, 1 patient with DHPR deficiency developed a pharmacoresistent epileptic encephalopathy with progressive neurological deterioration (despite adequate dopaminergic treatment), and 10 patients with DTDS showed a progressive neurodegenerative disease course with minimal response to drug intervention. Progression of hyperkinetic movements was commonly seen as part of disease evolution in patients with *GNAO1* (7/7) and *KMT2B* (15/17) variants and less frequently in patients with *FOXG1*‐ (6/17), *ATP1A3*‐ (2/6), *ADCY5*‐ (2/4), and *SGCE*‐related (3/30) disorders. Effective therapies included the ketogenic diet (*SLC2A1*; n = 4), flunarizine (*ATP1A3*; n = 4), and tetrabenazine (*GNAO1*; n = 2). Some patients with HMDs also responded partially or transiently to l‐dopa, benzodiazepines, trihexyphenidyl, carbamazepine, acetazolamide, and topiramate. Deep brain stimulation (DBS) of the internal globus pallidus was effective in 13 patients with drug‐resistant *SGCE*‐, *KMT2B*‐, and *GNAO1*‐related HMDs but did not show significant improvement in one 10‐year‐old boy with a severe *PDE10A*‐related HMD after 2‐year follow‐up (Table 1, Table S2 in Appendix S1).

## Discussion

Through this multicenter study, we have delineated the genetic landscape of complex HMDs, where diagnostic difficulty is frequently encountered, given the paucity of reliable blood/urine biomarkers, often nonspecific neuroimaging, and broad differential diagnosis. Complex HMD disorders are commonly misdiagnosed, for example, as “dyskinetic” or “dystonic” forms of acquired cerebral palsy.^3^, ^4^ Accurate clinical diagnosis is essential for prompt instigation of gene‐specific treatments, disease prognostication, and genetic counseling.

Despite the individual rarity of these conditions, we were able to characterize a large cohort of patients with HMDs of broad clinical and genetic heterogeneity. As a result, we provide a comprehensive analysis of motor semiology and associated clinical features in pediatric complex HMDs, together with video recorded material, that will be valuable for both diagnostic and educational purposes.

Our study highlights the increasing diagnostic value of multigene panels and whole‐exome/genome sequencing, which led to a diagnosis in almost one third of the cohort. The identification of several microdeletions encompassing disease‐causing genes (*FOXG1*, *KMT2B*, *NKX2*.*1*, *SGCE*) also highlights the diagnostic utility of aCGH for complex HMD.^5^, ^6^ Variants in a broad range of genes were identified, with different but interconnected cellular functions (Fig. 1). Genes involved in primary neurotransmitter disorders affect dopamine synthesis (*DDC*, *DHPR*, *PTPS*, *SPR*, and *TH*) and dopamine transport (*SLC6A3*). Mutations in *GNAO1* and other G protein subunits (*GNAL*), adenylyl cyclase (*ADCY5*), and cyclic nucleotide phosphodiesterase (*PDE10A*) disrupt the postsynaptic G protein–cAMP pathway axis and may impair neuromodulation or transduction of transmembrane signaling, presynaptic autoinhibitory effects, and altered neuronal excitability. Other HMD genes code for neuronal proteins with key biological roles, including Na^+^/K^+^‐ATPase transport, osmoregulation, and excitability (*ATP1A3*), transcriptional repressors involved in the promotion of neurogenesis and cortical neuronal differentiation (*FOXG1*), transcription factors essential for striatal development (*NKX2‐1*), dystrophin–glycoprotein complex that links the actin cytoskeleton to the extracellular matrix (*SGCE*), regulation of mitochondrial Ca^2+^ uptake and synaptic transmission (*MICU1*), glucose transport (*SLC2A1*), and posttranscriptional regulation of gene expression (*KMT2B*).

From a clinical perspective, pure HMD phenotypes (defined as those with a single manifesting HMD phenotype) were exceptional in our cohort of patients. We observed a pure dystonic motor semiology in patients with *KMT2B*‐ and neurotransmitter disorder–related HMDs, leading to gait difficulties in early childhood.^6^, ^7^ Also, a minority of patients with *NKX2‐1* defects showed a pure chorea phenotype. In contrast, most patients had a combination of two or more HMDs, or a complex movement disorder with both hyperkinetic and hypokinetic features, commonly known as dystonia‐plus syndromes. In some children with parkinsonism‐dystonia caused by biogenic amine defects, hypokinesia and bradykinesia may be severe and predominate over the HMD phenotype.^3^, ^8^

Chorea was the second most frequently identified HMD, evident in >50% patients with *ADCY5*, *ATP1A3*, *FOXG1*, *NKX2*.*1*, *SLC2A1*, *GNAO1*, and *PDE10A* variants.^9^, ^10^, ^11^, ^12^, ^13^, ^14^, ^15^ Chorea was mostly present in combination with dystonia and sometimes myoclonus. Myoclonus was characteristic of *SGCE* patients,^16^ but also observed in *ATP1A3* disease,^17^ *NKX2*.*1*,^13^, ^18^ *FOXG1*,^11^ *GNAO1*,^14^ *KMT2B*,^6^ *SLC2A1*, and some neurotransmitters defects,^19^, ^20^ usually in combination with chorea and dystonia. Myoclonus‐dystonia has also been reported in children with *ADCY5* and *GCH* ^21^ defects, but this combination was not observed in our cohort of patients. As previously reported, stereotypies were a key disease feature in *FOXG1* and *GNAO1* patients, always in combination with other HMDs.^11^, ^14^ Abnormal ocular, facial, and oromandibular movements were also important clues for diagnosis: eyelid myoclonus, ocular flutter, and oculogyric crises were exclusively observed in neurotransmitter diseases.^22^ Forehead wrinkling, facial grimacing, perioral muscle twitching, jaw dystonia, and tongue dyskinesias were frequently seen in children with generalized choreodystonia phenotypes because of *ADCY5*, *NKX2*.*1*, *GNAO1*, and *ATP1A3* mutations.^9^, ^10^, ^12^, ^14^

In our cohort, the majority had a generalized pattern of HMDs. Only a few patients showed a focal or segmental distribution, predominantly affecting the upper limbs and neck, with significantly later onset of the HMD. Genetic defects presenting in the first year of life were more likely to lead to both a generalized HMD pattern and also developmental delay, as observed in patients with neurotransmitter defects (with the exception of dominant *GCH1*‐related disease) and those with *ATP1A3*, *FOXG1*, *GNAO1*, *SLC2A1*, and *PDE10A* variants.^10^, ^11^, ^13^, ^14^, ^15^ Furthermore, in *NKX2*.*1*‐related HMDs, patients had delayed motor development and early hypotonia in infancy.^12^, ^18^ In addition to developmental delay, many patients in the cohort had other neurological and systemic features. Epilepsy was present in one quarter of our cohort, especially in patients with *FOXG1*, *GNAO1*, *ATP1A3*, and *SLC2A1* variants. The co‐occurrence of epilepsy and hyperkinetic phenotypes in genetic syndromes is increasingly recognized.^23^ Underlying mechanisms are not entirely clear but may be a consequence of the gene defect, as well as epigenetic phenomena, environmental factors, or the effect of motor evolution in the developing brain.^24^

Many complex genetic HMDs are also associated with disease‐specific paroxysmal exacerbations. Oculogyric crises appeared to be exclusive to patients with neurotransmitters defects.^3^, ^22^ Plegic attacks were identified in patients with *ATP1A3* variants and Glut1 deficiency.^13^, ^17^ More recently, acute hemiplegia has also been reported in patients with *ADCY5* variants.^25^ Within our series, status dystonicus was seen in patients with *GNAO1*, *KMT2B*, and *SLC6A3* mutations.^6^, ^14^, ^26^ Some paroxysmal episodes were caused by specific triggers. Exercise‐induced HMDs were seen in Segawa disease^27^ and Glut1 deficiency syndrome.^28^ Diurnal fluctuation, with improvement after sleep, was recorded in all neurotransmitter defects.^3^, ^22^ As previously reported, paroxysmal chorea during sleep and on wakening was seen in *ADCY5*‐related disease.^9^ We also identified several other triggers in our cohort, including fever, intercurrent illness, fatigue, emotional stress, temperature, purposeful movements, and fasting.

Complex genetic HMDs also significantly impact on DLAs. More than half of the cohort showed gait and speech impairment, and a significant proportion also showed difficulties in eating, hygiene, and dressing. It is likely that for some conditions (*FOXG1*, *GNAO1*), intellectual disability also contributed to functional impairment.^11^, ^14^ Gait impairment was a common finding in our cohort, with half of the patients (*GNAO1*, *DDC*, *PDE10A*, *SLC6A3*, and *FOXG1* defects) either unable to walk at all or requiring assistance for ambulation. In contrast, a mildly impaired but independent gait pattern was commonly observed in patients with drug‐responsive neurotransmitters defects (*GCH1*, *DHPR*, *SPR*, *TH*, and *PTPS*), nonprogressive chorea (*NKX2*.*1*, *ADCY5*), Glut1 syndrome (*SLC2A1*), and myoclonus‐dystonia (*SGCE*).

Standard investigations, including blood/urine tests and neuroimaging, are unyielding for the genetic defects included in our study. Rarely, subtle neuroimaging patterns can be present, including corpus callosum abnormalities and brain atrophy in *GNAO1* and *FOXG1* defects,^11^, ^29^ and subtle T2 low signal intensity within the globus pallidus in *KMT2B‐*dystonia.^6^ Interestingly, patients with heterozygous mutations in PDE10A show bilateral striatal necrosis, while those included in our study with biallelic variants have normal structural magnetic resonance imaging despite having low levels of PDE10A protein in the striatum.^15^ Our study highlights the merit of undertaking cerebrospinal fluid glucose and neurotransmitters, particularly to aid diagnosis of Glut1 deficiency and neurotransmitter defects (Fig. 2). In patients with suspected disorders of monoamine metabolism, urine sepiapterin or blood prolactin levels can also be measured; however, they are neither universally available nor sensitive/specific for diagnostic purposes. Although not disease specific, hypothyroidism was evident in *NKX2*.*1* disease^12^ and increased creatine kinase levels in patients with *MICU1* defects.^30^

Genetic characterization of patients with complex HMDs may facilitate selection of appropriate therapies. Many patients with neurotransmitter defects in our series showed a significant and sustained improvement with dopaminergic agents.^22^ However, our study confirms that both AADC deficiency and DTDS remain challenging, where novel treatment strategies, such as gene therapy, may have a role.^31^ Ketogenic diet improved motor performance and paroxysmal episodes in four of five patients with Glut1 deficiency.^28^ More recently, triheptanoin has been shown to dramatically reduce paroxysmal motor disorders and epileptic discharges in patients with Glut1 deficiency in open‐label pilot studies,^32^ but these results were not confirmed in a randomized, blinded, placebo‐controlled clinical trial, suggesting that triheptanoin is of limited therapeutic use.^33^

Recently, the Rare Movement Disorders Study Group of the International Parkinson and Movement Disorder Society designed an online survey to identify worldwide barriers for the genetic diagnosis of movement disorders. They found limited access to genetic testing in all countries compared with Europe and North America. Given these findings, it is important to emphasize that patients with early‐onset dystonia of unknown etiology should receive a trial with l‐dopa to exclude a possible defect in dopamine metabolism, regardless of the availability of genetic testing.^34^

DBS remains important for patients with medically intractable dystonia, and the contribution of genetic testing to outcome from DBS is increasingly recognized.^35^ In our cohort, DBS was effective for 13 patients with *KMT2B*‐, *SGCE*‐, and *GNAO1*‐related disease.^6^, ^36^, ^37^ DBS reduced the risk for life‐threatening hyperkinetic exacerbations in *GNAO1* patients. Marked improvement of choreoathetosis (albeit with only mild functional recovery) has also been reported in *ADCY5* disease after DBS.^9^

Although we studied a broad patient population from multiple centers, there are a number of study limitations. The lack of standardization of video footage may have affected HMD classification, although researchers were stringent in analyzing only videos where the whole body could be assessed. Furthermore, our strict inclusion criteria excluded a number of genetic HMDs, including those with isolated motor semiology (eg, DYT1 dystonia), as well as some metabolic diseases, and disorders where the HMD is a less prominent part of the clinical phenotype (eg, epileptic encephalopathies). This may have led to an overall ascertainment bias when considering complex genetic HMDs, although our deliberate aim was to focus on better delineating complex HMD disorders where the HMD was the main phenotype without highly predictive blood, urine, or radiological biomarkers. Finally, it is important to emphasize that the motor features identified in our cohort are derived from a very small subset of individuals with each genetic defect; therefore, the spectrum of movement disorders analyzed in this study does not fully represent the breath of HMDs that may be associated with a particular gene.

In conclusion, detailed clinical assessment and careful classification of the HMD semiology is key to diagnosing complex genetic HMDs. If neuroimaging, blood, and urine neurometabolic testing are unyielding, cerebrospinal fluid analysis and targeted neurogenetic investigations should be promptly undertaken. In the future, better understanding of the underlying disease mechanisms will no doubt facilitate the development of precision treatment strategies for these disorders.

## Author Roles

B.P.‐D. and M.A.K. conceived, organized, and executed the project. They also drafted the manuscript. K.G., J.D.O.‐E., A.M., A.M.‐G., F.R.D., K.B., and E.M. assisted in data analysis and contributed to writing sections of the manuscript. A.P., J.N., S.S.M., M.S., F.M., P.M., J.U., P.V., E.S., R.G., J.C., S.Y., E.D.G., R.C.D., R.P., K.J.P., and V.L. provided clinical data for the project and reviewed and critiqued the manuscript.

## Financial Disclosures

## Supporting information


**Appendix S1** Supporting information.

**Video S1** Combined Hyperkinetic Movement Disorders. Patient 40 (*FOXG1*). In the video, she is lying in the hospital bed. She presents complex hyperkinetic movements that include choreo‐dystonia of all extremities, along with stereotypies of both hands. Patient 50 (*GNAO1*). Two videos of the same patient are presented: in the first one, he is sitting in his baby stroller and in the second, he is lying in bed. In both videos, prominent and generalized choreo‐dystonic movements are observed. Stereotypies of the hands are present, as well as intermittent involuntary mouth opening. The movements are continuous and in the second video there is an impression of discomfort. Patient 83 (*PDE10A*). In the first part of the video, he transfers from his wheelchair to the floor. He presents brief, generalized, random choreic movements affecting the face, the four limbs and the axial muscles, with dystonic postures of the left upper extremity at the beginning of the video. In the second part of the video, he is moving on the floor with flexed lower extremities and extended upper extremities on both sides of the trunk. Finally, in the last part of the video the patient is seated. There are axial dystonic movements causing torticollis, trunk hyperextension and transient truncal instability.

**Video S2** Dystonia. Patient 13 (*ADCY5*). The video shows the patient standing. He has cervical dystonia that causes laterocollis to the right, with limited head rotation to the left. His laterocollis is persistent even when asked him to raise his arms. Unlike his brother (Patient 2), he does not have associated choreiform movements or perioral dyskinesias. Patient 85 (*SGCE*). In the video, the patient is walking. He has walking induced focal dystonia with left arm elevation and trunk hyperextension. Dystonia becomes more prominent as the patient increases the speed of walking. Patient 136 *(KMT2B)*. The patient appears standing with assistance of his mum, and then sitting in his wheelchair. In the first part of the video, there is evidence of bilateral dystonic foot postures. He suffers from intermittent mouth opening, due to oromandibular dystonia. During the finger‐finger test, dystonia is evident, predominating in the left upper limb. During foot stomping maneuver, foot dystonia is observed, as well as involuntary mouth opening. Patient 127 (*GNAO1*). The patient is walking. He has generalized dystonic movements that affect gait stability. Patient 86 (*SGCE*). He has gait dystonia that partially disappears when he walks backwards and when his gait is assisted by his father. At the end of the video, sudden left foot inversion causes him to fall.

**Video S3** Chorea. Patient 11 (*ADCY5*). The video shows the patient sitting in a high back chair. She has poor head control due to axial hypotonia, as well as continuous choreoathetoid movements in both hands, and myoclonic jerks affecting the trunk and the four limbs. Dystonic left leg posturing is visible. Patient 12 (*ADCY5*). The patient is sitting on the bed. He has evidence of fast irregular choreiform movements affecting mainly the upper limbs distally, but also the face and the tongue. The choreiform movements are exacerbated with specific voluntary actions, such as arm extension, tongue protrusion and facial movements. Patient 53 (*NKX2.1*). In this video, she is sitting without support on a stretcher. She has choreiform movements during the finger‐finger test and also when extending the arms forward. These movements are seen distally in the hands and proximally in the shoulders as well as the abdomen. Choreiform movements are observed during tongue protrusion. Patient 56 (*NKX‐2.1*). In the video, the child is seated and then he appears walking. He presents with choreiform movements at rest, which predominate in the proximal region of the upper extremities and in the trunk. He also exhibits some hyperkinetic movements in his fingers. While walking, there is generalized chorea and a dystonic gait pattern, which do not majorly impact on gait stability. Patient 15 (*ATP1A3*). The child is sitting independently with arms extended forward. He has mild choreiform movements of the upper and lower extremities. An axial myoclonic jerk is also observed.

**Video S4** Myoclonus. Patient 87 (*SGCE*). The patient is walking, and demonstrates several abrupt brief myoclonic jerks that affect the trunk, head, upper and lower extremities moreso on the left side of the body. These myoclonic jerks have a moderate impact on gait stability. Patient 88 (*SGCE*). The patient is sitting on the floor playing with a cell phone. He has myoclonic jerks in the lower extremities together with dystonic posturing of both legs. He shows trunk instability as a result of these myoclonic movements. Patient 104 (*SLC2A1*). The patient is sitting. The finger‐nose maneuver triggers a sudden, violent and brief myoclonic jerk.

**Video S5** Infantile parkinsonism–dystonia. Patient 6 (*AADC*). In the video, the patient is sitting in her baby stroller. There is a paucity of spontaneous movement with decreased blinking, facial hypomimia and ptosis. Patient 113 (*TH*). In the video, the patient is lying on the bed. There is severe bradykinesia, evident when he tries to lower his right upper limb. He also has hypomimia and sialorrhea, bilateral coarse tremor of the upper limbs and rigidity with clenched fists.

## Data Availability

Data available on request due to privacy/ethical restrictions
