# Supplementary material for: The Genetic Landscape of Complex Childhood‐Onset Hyperkinetic Movement Disorders
Source: Mov Disord. 2022 Aug 25;37(11):2197–209. doi: 10.1002/mds.29182 (PMC9804670; doi:10.1002/mds.29182)
Supplement: Supplementary file 1 — Appendix S1 Supporting information. [file MDS-37-2197-s004.docx]

**Supplementary Data:**

**The Genetic Landscape of Complex Childhood-Onset Hyperkinetic Movement Disorders**

**^1,2 *^Belén Pérez-Dueñas**, MD, PhD, ^3,4^**Kathleen Gorman**, MD, ^2^**Juan D Ortigoza-Escobar**, MD, PhD, ^1^**Alfons Macaya,** MD, PhD, ^5^**Federica R Danti**, MD, ^3^**Katy Barwick** PhD, ^1^**Anna Marcé-Grau**, PhD, ^3,4^**Apostolos Papandreou**, MD, ^6^**Joanne Ng**, MD, PhD, ^3^**Esther Meyer**, PhD, ^7^**Shekeeb S Mohammad**, FRACP, PhD, ^8^**Martin Smith MD, PhD,** ^3,4^**Francesco Muntoni** MD, PhD, ^4^**Pinki Munot**, MD, ^9^**Johanna Uusimaa**, MD, PhD, ^9^**Päivi Vieira,** MD, PhD, ^10^**Eammon Sheridan,** MD, ^11^**Renzo Guerrini** MD, FRCP, ^12^**Jan Cobben,** MD, PhD, ^13^**Sanem Yilmaz**, MD, ^14^**Elisa De Grandis,** MD, PhD, ^15,^**Russell C Dale,** MD, PhD, ^16^**Roser Pons**, MD, PhD, ^17^**Kathryn J Peall**, MD, PhD, ^5^**Vincenzo Leuzzi**, MD, PhD, ^3,4^***Manju A Kurian**, MD, PhD.

**Supplementary Methods**

**List of Participating Study Centres**

1. Department of Neurology, Great Ormond Street Hospital, London, UK
2. Department of Paediatrics, Child Neurology and Psychiatry, Sapienza University of Rome, Italy
3. Institute for Neuroscience and Muscle Research, Children's Hospital at Westmead, University of Sydney, Australia
4. First Department of Pediatrics, Agia Sofia Children's Hospital, Athens, Greece
5. Centre for Neuropsychiatric Genetics and Genomics, Institute of Psychological Medicine and Clinical Neurosciences, Cardiff, UK
6. Department of Pediatric Neurology, Hospital Vall d’Hebrón, Barcelona, Spain
7. Department of Pediatric Neurology, Sant Joan de Déu Hospital, Barcelona, Spain
8. Child Neuropsychiatry Unit, Istituto Giannina Gaslini, Genova, Italy
9. Department of Paediatric Neurology, John Radcliffe Hospital, Oxford, UK
10. Dubowitz Neuromuscular Centre, UCL Institute of Child Health and Great Ormond Street Hospital for Children, London, UK
11. Department of Children and Adolescents, Medical Research Center, Oulu University Hospital, Finland
12. Clinical & Paediatric Genetics, St George's Hospital & University of London
13. Pediatric Neurology, Neurogenetics and Neurobiology Unit, Meyer Children's Hospital, University of Florence
14. Division of Child Neurology, Ege University Medical Faculty, Turkey

**Ethical approval and consent**

1. UK Health Research Authority (HRA) approval IRAS 240756: Genetic Landscape of Complex Childhood Hyperkinetic Movement Disorders
2. UK Health Research Authority (HRA) approval IRAS 146495: Move Wales: Welsh Movement Disorders Research Network.
3. The Ethics Committee at Vall d’Hebron University Hospital in Barcelona (PR(AG)430/2019).
4. Paediatric Ethics Committee of the Tuscany Region.

**The functional impact of HMD on daily living activities (DLA)**

The functional impact of HMD on daily living activities (DLA) was scored as follows:

- Gait impairment (patients > 2 years)

0 = normal gait

1 = independent but slow gait, frequent falls and/or imbalance

2 = requires an assistive device for safe walking

3= cannot walk at all or only with another person’s assistance

- Speech (patients > 3 years)

0= normal

1= impaired

- Eating and swallowing

0 = normal

1 = impaired

- Daily living activities requiring fine motor tasks (i.e. using cutlery, dressing, hygiene for patients > 5 years) were classified as either independent or dependent.

**Supplementary Results**

**Supplementary Table 1 :**

Complete dataset for 140 patients with complex genetic HMD.

**Supplementary Table 2:**

Functional impact, associated clinical features and long-term outcome in patients with complex genetic HMD

**Supplementary Table 3:**

Impact of complex genetic HMD on daily living activities

**Supplementary Table 4:**

Complex genetic HMD presenting with paroxysmal events or exacerbations, and associated trigger

**Supplementary References (linked to Supplementary Table 1)**

1. Fornarino S, Stagnaro M, Rinelli M, et al. Paroxysmal features responding to flunarizine in a child with rapid-onset dystonia-parkinsonism. Neurology. 2014; 82:2037-8
2. Leuzzi V, Mastrangelo M, Polizzi A, Artiola C, van Kuilenburg AB, Carducci C, Ruggieri M, Barone R, Tavazzi B, Abeling NG, Zoetekouw L, Sofia V, Zappia M, Carducci C. Report of two never treated adult sisters with aromatic L-amino Acid decarboxylase deficiency: a portrait of the natural history of the disease or an expanding phenotype? JIMD Rep. 2015;15:39-45.
3. Papandreou A, Schneider RB, Augustine EF, et al. Delineation of the movement disorders associated with FOXG1 mutations. Neurology. 2016; 86:1794-800
4. Dale RC, Melchers A, Fung VS, et al. Familial paroxysmal exercise-induced dystonia: atypical presentation of autosomal dominant GTP-cyclohydrolase 1 deficiency. Dev Med Child Neurol. 2010; 52: 583-6
5. Leuzzi V, Carducci C, Carducci C, et al. Autosomal dominant GTP-CH deficiency presenting as a dopa-responsive myoclonus-dystonia syndrome. Neurology. 2002; 59(8):1241-3
6. Yilmaz S, Turhan T, Ceylaner S, et al. Excellent response to deep brain stimulation in a young girl with GNAO1-related progressive choreoathetosis. Childs Nerv Syst. 2016; 32:1567-8
7. Danti FR, Galosi S, Romani M, et al. GNAO1 encephalopathy: Broadening the phenotype and evaluating treatment and outcome. Neurol Genet. 2017; 3:e143
8. Menke LA, Engelen M, Alders M, Odekerken VJ, Baas F, Cobben JM. Recurrent GNAO1 Mutations Associated With Developmental Delay and a Movement Disorder. J Child Neurol. 2016 Dec;31(14):1598-1601
9. Meyer E, Carss KJ, Rankin J, et al. Mutations in the histone methyltransferase gene KMT2B cause complex early-onset dystonia. Nat Genet. 2017; 49:223-237
10. Logan CV, Szabadkai G, Sharpe JA, et al. Loss-of-function mutations in MICU1 cause a brain and muscle disorder linked to primary alterations in mitochondrial calcium signalling. Nat Genet. 2014; 46:188-93
11. Peall KJ, Lumsden D, Kneen R, et al. Benign hereditary chorea related to NKX2.1: expansion of the genotypic and phenotypic spectrum. Dev Med Child Neurol. 2014; 56: 642-648
12. Fons C, Rizzu P, Garcia-Cazorla A, et al. TITF-1 gene mutation in a case of sporadic non-progressive chorea. Response to levodopa treatment. Brain Dev. 2012; 34:255-7
13. Dale RC, Grattan-Smith P, Nicholson M, et al. Microdeletions detected using chromosome microarray in children with suspected genetic movement disorders: a single-centre study. Dev Med Child Neurol. 2012; 54:618-23
14. McMichael G, Haan E, Gardner A, et al. NKX2-1 mutation in a family diagnosed with ataxic dyskinetic cerebral palsy. Eur J Med Genet. 2013; 56:506-9
15. Diggle CP, Sukoff Rizzo SJ, et al. Biallelic Mutations in PDE10A Lead to Loss of Striatal PDE10A and a Hyperkinetic Movement Disorder with Onset in Infancy. Am J Hum Genet. 2016; 98: 735-43
16. Leuzzi V, Carducci CA, Carducci CL, Pozzessere S, Burlina A, Cerone R, Concolino D, Donati MA, Fiori L, Meli C, Ponzone A, Porta F, Strisciuglio P, Antonozzi I, Blau N. Phenotypic variability, neurological outcome and genetics background of 6-pyruvoyl-tetrahydropterin synthase deficiency. Clin Genet. 2010 Mar;77(3):249-57
17. Peall KJ, Smith DJ, Kurian MA, et al. [SGCE mutations cause psychiatric disorders: clinical and genetic characterization.](https://www.ncbi.nlm.nih.gov/pubmed/23365103) Brain. 2013; 136:294-303
18. Pérez-Dueñas B, Prior C, Ma Q, et al. Childhood chorea with cerebral hypotrophy: a treatable GLUT1 energy failure syndrome. Arch Neurol. 2009; 66:1410-4
19. Kurian MA, Zhen J, Cheng SY, et al. Homozygous loss-of-function mutations in the gene encoding the dopamine transporter are associated with infantile parkinsonism-dystonia. J Clin Invest. 2009; 119:1595-603
20. Kurian MA, Li Y, Zhen J et al. Clinical and molecular characterisation of hereditary dopamine transporter deficiency syndrome: an observational cohort and experimental study. Lancet Neurol. 2011; 10: 54-62
21. Ng J, Zhen J, Meyer E, Erreger K, et al. Dopamine transporter deficiency syndrome: phenotypic spectrum from infancy to adulthood. Brain. 2014;137(Pt 4):1107-19
22. Neville BG, Parascandalo R, Farrugia R et al. Sepiapterin reductase deficiency: a congenital dopa-responsive motor and cognitive disorder. Brain. 2005; 128:2291-6
23. Leuzzi V, Carducci C, Tolve M, et al. Very early pattern of movement disorders in sepiapterin reductase deficiency. Neurology. 2013; 81:2141-2
24. Pons R, Syrengelas D, Youroukos S, et al. Levodopa-induced dyskinesias in tyrosine hydroxylase deficiency. Mov Disord. 2013; 28:1058-63
25. Giovanniello T, Leuzzi V, Carducci C et al. Tyrosine hydroxylase deficiency presenting with a biphasic clinical course. Neuropediatrics. 2007; 38:213-5
26. Dionisi-Vici C, Hoffmann GF, Leuzzi V, et al. Tyrosine hydroxylase deficiency with severe clinical course: clinical and biochemical investigations and optimization of therapy. J Pediatr. 2000; 136:560-2

| **Supplementary Table 1:** Complete dataset for 140 patients with complex genetic HMD. | | | | | | | | | | | | |
| --- | --- | --- | --- | --- | --- | --- | --- | --- | --- | --- | --- | --- |
| **Patient number in the videos** | **Gene** | **OMIM-Gene related phenotype** | **Mutation** | **Effect on Protein** | **Inheritance Pattern** | **Method** | **Age at onset of HMD (years)** | **Age at last assessment (years)** | **Sex** | **Ethnicity** | **Consanguinity** | **Previously published (Suppl. Reference)** |
| 11 | *ADCY5* | #606703 - Familial dyskinesia with facial myokymia | c.1253G>A | p.Arg418Gln | Dominant de novo | WES | 0,75 | 9 | Female | White (European) | No | No |
| 12 | *ADCY5* | #606703 - Familial dyskinesia with facial myokymia | c.1252C>T | p.Arg418Trp | Dominant de novo | Single gene testing | 0,75 | 4 | Female | White (European) | No | No |
|  | *ADCY5* | #606703 - Familial dyskinesia with facial myokymia | c.3086T>G | p.Met1029Arg | Dominant inherited | Single gene testing | 1,5 | 11 | Male | White (European) | No | No |
| 13 | *ADCY5* | #606703 - Familial dyskinesia with facial myokymia | c.3086T>G | p.Met1029Arg | Dominant inherited | Single gene testing | 2,5 | 3 | Male | White (European) | No | No |
|  | *ATP1A3* | #104290 - Alternating Hemiplegia of Childhood | c.2443G>A | p.Glu815Lys | Dominant de novo | Single gene testing | 0,6 | 9,75 | Female | White (European) | No | No |
| 15 | *ATP1A3* | #104290 - Alternating Hemiplegia of Childhood | c.998G>T | p.Cys333Phe | Dominant de novo | Single gene testing | 0,08 | 13,33 | Male | White (European) | No | No |
|  | *ATP1A3* | #182350 -Sodium-potasium-ATPase spectrum disorder (intermediate phenotype) | c.2267G>A | p.Arg756His | Dominant de novo | Single gene testing | 0,75 | 15,60 | Female | White (European) | No | 1 |
|  | *ATP1A3* | #128235 - Rapid-onset dystonia-parkinsonism (DYT12) | c.2266 C>T | p.Arg756Cys | Dominant de novo | Single gene testing | 0,6 | 8 | Male | White (European) | No | No |
|  | *ATP1A3* | #104290 - Alternating Hemiplegia of Childhood | c.2401G>A | p.Asp801Asn | Dominant de novo | Single gene testing | 0,4 | 29,9 | Female | White (European) | No | No |
|  | *ATP1A3* | #128235 - Rapid-onset dystonia-parkinsonism (DYT12) | c.2267G>A | p.Arg756His | Dominant inherited | Single gene testing | 4 | 14 | Male | White (European) | No | No |
|  | *DDC* | #608643 - Aromatic L-amino acid decarboxylase (AADC) deficiency | c.328G>A/  c.1140G>A | p.Ala110Thr/  p.Lys380= | Recessive | CSF/Enzyme assay then single gene testing | 0,4 | 5 | Male | White (European) | No | No |
|  | *DDC* | #608643 - Aromatic L-amino acid decarboxylase (AADC) deficiency | c.1073G>A/  c.73G>A | p.Arg358His/  p.Glu25Lys | Recessive | CSF/Enzyme assay then single gene testing | 0,1 | 7 | Male | White (European) | No | No |
|  | *DDC* | #608643 - Aromatic L-amino acid decarboxylase (AADC) deficiency | c.1039C>G Homozygous | p.Arg347Gly | Recessive | CSF/Enzyme assay then single gene testing | 0,02 | 0,6 | Male | Asian (Pakistani) | Yes -distally related cousins | No |
|  | *DDC* | #608643 - Aromatic L-amino acid decarboxylase (AADC) deficiency | c.315G>C/  c.73G>A | p.Trp105Cys/  p.Glu25Lys | Recessive | CSF/Enzyme assay then single gene testing | 0,25 | 2 | Female | White (European) | No | No |
| 6 | *DDC* | #608643 - Aromatic L-amino acid decarboxylase (AADC) deficiency | c.476C>T/  c.175G>A | p.Ala159Val/  p.Asp59Asn | Recessive | CSF/Enzyme assay then single gene testing | 0,08 | 2 | Female | White (European) | No | No |
|  | *DDC* | #608643 - Aromatic L-amino acid decarboxylase (AADC) deficiency | c.799T>C homozygous | p.Trp267Arg | Recessive | Single gene testing | 0,6 | 1,3 | Female | White (European) | No | No |
|  | *DDC* | #608643 - Aromatic L-amino acid decarboxylase (AADC) deficiency | c.105delC/  c.710T>C | p.Tyr37Thrfs*5/  p. Phe237Ser | Recessive | Single gene testing | 0 | 22 | Female | White (European) | No | 2 |
|  | *DDC* | #608643 - Aromatic L-amino acid decarboxylase (AADC) deficiency | c.105delC/  c.710 T>C | p.Tyr37Thrfs*5/  p. Phe237Ser | Recessive | Single gene testing | 0 | 32 | Female | White (European) | No | 2 |
|  | *DDC* | #608643 - Aromatic L-amino acid decarboxylase (AADC) deficiency | c.749C>T homozygous | p.Ser250Phe | Recessive | Single gene testing | 0,5 | 11 | Male | White (European) | Yes - first cousins | No |
|  | *DHPR/*  *QDPR* | #261630 - Infantile parkinsonism- dystonia-BH4 deficiency-hyperphenylalaninaemia | c.661C>T/  c.609dupA | p.Arg221Ter/  p.Pro204Thrfs*7 | Recessive | Multigene panel | 0 | 1 | Female | White (European) | No | No |
|  | *DHPR/*  *QDPR* | #261630 - Infantile parkinsonism- dystonia-BH4 deficiency-hyperphenylalaninaemia | c.466G>A/  c.609dupA | p.Ala156Thr/  p.Pro204Thrfs*7 | Recessive | Multigene panel | 1 | 1 | Male | White (European) | No | No |
|  | *DHPR/*  *QDPR* | #261630 - Infantile parkinsonism- dystonia-BH4 deficiency-hyperphenylalaninaemia | c.547delG homozygous | p.Val183Leufs*7 | Recessive | Single gene testing | 0,2 | 0,5 | Male | Asian (Pakistani) | Yes - first cousins | No |
|  | *DHPR/*  *QDPR* | #261630 - Infantile parkinsonism- dystonia-BH4 deficiency-hyperphenylalaninaemia | c.41T>C homozygous | p.Leu14Pro | Recessive | Single gene testing | 2 | 10 | Female | White (European) | No | No |
|  | *FOXG1* | #613454 - Rett syndrome, congenital variant | c.651C>G | p.Tyr217* | Dominant de novo | Multigene panel | 0 | 5 | Male | White (European) | No | 3 |
|  | *FOXG1* | #613454 - Rett syndrome, congenital variant | 1.1 Mb del chr14:28552713-29655317 | Gene deletion | Dominant de novo | arrayCGH | 0,3 | 3,5 | Female | White (European) | No | 3 |
|  | *FOXG1* | #613454 - Rett syndrome, congenital variant | c.460dupG | p.Glu154Glyfs*301 | Dominant de novo | Multigene panel | 1 | 6,3 | Male | White (European) | No | 3 |
|  | *FOXG1* | #613454 - Rett syndrome, congenital variant | c.515_577del63 | p.Gly172_Met192del | Dominant - likely parental gonadal mosaicism | Multigene panel | 1 | 6,8 | Female | White (European) | No | 3 |
|  | *FOXG1* | #613454 - Rett syndrome, congenital variant | c.586C>T | p.Gln196* | Dominant de novo | Multigene panel | 0,75 | 3,6 | Male | White (European) | No | 3 |
|  | *FOXG1* | #613454 - Rett syndrome, congenital variant | c.460dupG | p.Glu154Glyfs*301 | Dominant - likely parental gonadal mosaicism | Multigene panel | 0,5 | 25 | Female | White (European) | No | 3 |
|  | *FOXG1* | #613454 - Rett syndrome, congenital variant | c.460dupG | p.Glu154Glyfs*301 | Dominant - likely parental gonadal mosaicism | Multigene panel | 0,25 | 22 | Female | White (European) | No | 3 |
|  | *FOXG1* | #613454 - Rett syndrome, congenital variant | c.222_223dupGC | p.Pro75Argfs*118 | Dominant de novo | Multigene panel | 0 | 8 | Male | White (European) | No | 3 |
|  | *FOXG1* | #613454 - Rett syndrome, congenital variant | c.460dupG | p.Glu154Glyfs*301 | Dominant de novo | Multigene panel | 1 | 5 | Female | White (European) | No | 3 |
|  | *FOXG1* | #613454 - Rett syndrome, congenital variant | c.460dupG | p.Glu154Glyfs*301 | Dominant de novo | Multigene panel | 1 | 5 | Female | White (European) | No | 3 |
|  | *FOXG1* | #164874 - Forkhead Box G1 - mild variant | c.695A>G | p.Asn232Ser | Dominant de novo | Multigene panel | ? | 17 | Male | White (European) | No | 3 |
|  | *FOXG1* | #613454 - Rett syndrome, congenital variant | c.735delC | p.Tyr246Thrfs*80 | Dominant de novo | Multigene panel | ? | 7 | Male | White (European) | No | 3 |
|  | *FOXG1* | #613454 - Rett syndrome, congenital variant | c.460dupG | p.Glu154Glyfs*301 | Dominant de novo | Multigene panel | ? | 17 | Female | White (European) | No | 3 |
|  | *FOXG1* | #613454 - Rett syndrome, congenital variant | 2kb deletion at Chr14q12 (coordinates not available) | Gene deletion | Dominant de novo | arrayCGH |  | 8 | Female | White (European) | No | 3 |
|  | *FOXG1* | #164874 - Forkhead Box G1 - mild variant | c.572T>G | p.Met191Arg | Dominant - maternal somatic mosaicism | Multigene panel | ? | 10 | Male | White (European) | No | 3 |
|  | *FOXG1* | #164874 - Forkhead Box G1 - mild variant | c.572T>G | p.Met191Arg | Dominant - maternal somatic mosaicism | Multigene panel | ? | 13 | Female | White (European) | No | 3 |
| 40 | *FOXG1* | #164874 - Forkhead Box G1 - mild variant | c.610C>G | p.Leu204Val | Dominant de novo | Multigene panel | 0,3 | 2,6 | Female | White (European) | No | No |
|  | *GCH1* | #128230 - Dopa responsive dystonia_Autosomal Dominant guanosine-triphosphate-cyclohydrolase deficiency | c.677_678delTG | p.Val226Aspfs*23 | Dominant inherited | Single gene testing | 1,4 | 5 | Female | White (European) | No | No |
|  | *GCH1* | #128230 - Dopa responsive dystonia_Autosomal Dominant guanosine-triphosphate-cyclohydrolase deficiency | c.250G>T | p.Glu84* | Dominant inherited | Single gene testing | 15 | 41 | Female | White (European) | No | 4 |
|  | *GCH1* | #128230 - Dopa responsive dystonia_Autosomal Dominant guanosine-triphosphate-cyclohydrolase deficiency | c.646C>T | p.Arg216* | Dominant inherited | Single gene testing | 2 | 4 | Male | White (European) | No | No |
|  | *GCH1* | #128230 - Dopa responsive dystonia_Autosomal Dominant guanosine-triphosphate-cyclohydrolase deficiency | c.250G>T | p.Glu84* | Dominant inherited | Single gene testing | 7 | 10 | Male | White (European) | No | 4 |
|  | *GCH1* | #128230 - Dopa responsive dystonia_Autosomal Dominant guanosine-triphosphate-cyclohydrolase deficiency | c.631_632delAT | p.Met211Valfs*38 | Dominant inherited | Single gene testing | 9 | 11 | Female | White (European) | No | No |
|  | *GCH1* | #128230 - Dopa responsive dystonia_Autosomal Dominant guanosine-triphosphate-cyclohydrolase deficiency | c.285_286delCT | p.Trp96Glufs*27 | Dominant inherited | Single gene testing | 2 | 6 | Female | White (European) | No | No |
|  | *GNAO1* | #617493 - Neurodevelopmental disorder with involuntary movement | c.698A>C | p.Gln233Pro | Dominant de novo | Single gene testing | 2 | 5,5 | Female | White (European) | No | 6 |
|  | *GNAO1* | #617493 - Neurodevelopmental disorder with involuntary movements | c.139A>G | p.Ser47Gly | Dominant de novo | WES | 0,33 | 0,66 | Male | Mixed (Asian/European) | No | 7 |
|  | *GNAO1* | #617493 - Neurodevelopmental disorder with involuntary movements | c.625C>T | p.Arg209Cys | Dominant de novo | WGS | 8,91 | 8,91 | Male | White (European) | No | 7 |
| 50 | *GNAO1* | #617493 - Neurodevelopmental disorder with involuntary movements | c.625C>T | p.Arg209Cys | Dominant de novo | Multigene panel | 0,58 | 3,5 | Male | White (European) | No | 7 |
|  | *GNAO1* | #617493 - Neurodevelopmental disorder with involuntary movement | c.737A>G | p.Glu246Gly | Dominant de novo | WES | 2 | 14 | Male | White (European) | No | 7 |
|  | *GNAO1* | #617493 - Neurodevelopmental disorder with involuntary movements | c.626G>A | p.Arg209His | Dominant de novo | WES | 1,5 | 3 | Male | White (European) | No | 8 |
| 127 | *GNAO1* | #617493 - Neurodevelopmental disorder with involuntary movements | c.625C>T | p.Arg209Cys | Dominant de novo | WES | 6,58 | 1 | Male | White (European) | No | No |
|  | *KMT2B* | #617284 - Dystonia 28, childhood-onset | Del chr19: 35608666-36233508 | Gene deletion | Dominant de novo | arrayCGH | 4 | 9 | Male | White (European) | No | 9 |
|  | *KMT2B* | #617284 - Dystonia 28, childhood-onset | Del chr19: 35197252-38140100 | Gene deletion | Dominant de novo | arrayCGH | 7 | 13 | Female | White (European) | No | 9 |
|  | *KMT2B* | #617284 - Dystonia 28, childhood-onset | Del chr19: 34697740-37084510 | Gene deletion | Dominant (parental status not known) | arrayCGH | 2,5 | 8 | Male | White (European) | No | 9 |
|  | *KMT2B* | #617284 - Dystonia 28, childhood-onset | Del chr19: 36191100-36376860 | Gene deletion | Dominant de novo | arrayCGH | 4 | 8 | Female | White (European) | No | 9 |
|  | *KMT2B* | #617284 - Dystonia 28, childhood-onset | Del chr19: 35414997-37579142 | Gene deletion | Dominant de novo | arrayCGH | 4 | 17 | Female | White (European) | No | 9 |
|  | *KMT2B* | #617284 - Dystonia 28, childhood-onset | Del chr19: 35967904-37928373 | Gene deletion | Dominant de novo | arrayCGH | 4 | 14 | Male | White (European) | No | 9 |
|  | *KMT2B* | #617284 - Dystonia 28, childhood-onset | c.402dup | p.Ser135GLnfs*23 | Dominant de novo | WGS | 6 | 25 | Female | White (European) | No | 9 |
| 136 | *KMT2B* | #617284 - Dystonia 28, childhood-onset | c.3143_3149del | p.Gly1048Glufs*132 | Dominant de novo | WGS | 8 | 18 | Male | White (European) | No | 9 |
|  | *KMT2B* | #617284 - Dystonia 28, childhood-onset | c.6515_6518  delinsCCCAA | p.Val2172Alafs*11 | Dominant de novo | WES | 1 | 17 | Male | White (European) | No | 9 |
|  | *KMT2B* | #617284 - Dystonia 28, childhood-onset | c.4955G>A | p.Gly1652Asp | Dominant de novo | WES | 6 | 11 | Male | White (European) | No | 9 |
|  | *KMT2B* | #617284 - Dystonia 28, childhood-onset | c.4986C>A | p.Phe1662Leu | Dominant inherited (mother) | WGS | 5 | 20 | Female | White (European) | No | 9 |
|  | *KMT2B* | #617284 - Dystonia 28, childhood-onset | c.5114G>A | p.Arg1705Gln | Dominant de novo | Single gene testing | 3 | 8 | Male | White (European) | No | 9 |
|  | *KMT2B* | #617284 - Dystonia 28, childhood-onset | c.5342T>C | p.Leu1781Pro | Dominant de novo | Single gene testing | 8 | 19 | Female | White (European) | No | 9 |
|  | *KMT2B* | #617284 - Dystonia 28, childhood-onset | c.8021T>C | p.Ile2674Thr | Dominant inherited (mother) | Single gene testing | 9 | 19 | Female | White (European) | No | 9 |
|  | *KMT2B* | #617284 - Dystonia 28, childhood-onset | c.3528+2T>A | Predicted splicing effect - donor site lost | Dominant (parental status not known) | WES | 4 | 40 | Male | White (European) | No | 9 |
|  | *KMT2B* | #617284 - Dystonia 28, childhood-onset | c.5284C>T | p.Arg1762Cys | Dominant de novo | WES | 6 | 27 | Female | White (European) | No | 9 |
|  | *MICU1* | #615673 - Myopathy with extrapyramidal signs | c.1078-1G>C | Predicted splicing effect - cryptic acceptor site activated | Recessive | WES | 3 | 20 | Female | African | No | 10 |
|  | *MICU1* | #615673 - Myopathy with extrapyramidal signs | c.1078-1G>C | Predicted splicing effect - cryptic acceptor site activated | Recessive | WES | 2 | 14 | Female | African | No | 10 |
|  | *NKX2-1* | #118700 - Benign Hereditary Chorea | c.1204T>A | p.*402Argext*63 | Dominant inherited | Single gene testing | 1 | 18 | Female | White (European) | No | 11 |
|  | *NKX2-1* | #118700 - Benign Hereditary Chorea | Del chr14: 36924171-37283221; including *SFTA3, NKX2-1, BX161496, NKX2.8, PAX9, SLC25A21* | Whole gene deletion | Dominant de novo | arrayCGH | 7 | 11 | Female | White (European) | No | 11 |
|  | *NKX2-1* | #118700 - Benign Hereditary Chorea | c.739_746del | p.Lys247Glyfs*189 | Dominant de novo | Single gene testing | 3 | 10 | Female | White (European) | No | No |
|  | *NKX2-1* | #118700 - Benign Hereditary Chorea | c.463+1G>A | Predicted splicing effect - donor site lost | Dominant de novo | Single gene testing | 1,6 | 9 | Female | White (European) | No | 12 |
| 53 | *NKX2-1* | #118700 - Benign Hereditary Chorea | c.*1597_*1598insG | 3'UTR alteration - Predicted splicing effect | Dominant inherited | Single gene testing | 2 | 4.5 | Male | White (European) | No | No |
| 56 | *NKX2-1* | #118700 - Benign Hereditary Chorea | Del chr14:28552713-29655317 ; including *NFKBIA, INSM2, RALGAPA1, BRMS1L, MBIP, SFTA3, NKX2-1, NKX2-8, PAX9, SLC25A21, MIPOL1, FOXA1, TTC6, SSTR1, CLEC14A* | whole gene deletion | Dominant de novo | arrayCGH | 1,5 | 9 | Male | Mixed (Asian/Australian) | No | 13 |
|  | *NKX2-1* | #118700 - Benign Hereditary Chorea | c.84_90del | p.Met29Alafs*40 | Dominant inherited | WES | 3 | 14 | Male | White (European) | No | 14 |
|  | *NKX2-1* | #118700 - Benign Hereditary Chorea | c.84_90del | p.Met29Alafs*40 | Dominant inherited | WES | 3 | 15 | Female | White (European) | No | 14 |
| 80 | *PDE10A* | #616921 - Dyskinesia, limb and orofacial, infantile-onset | c.346G>C | p.Ala116Pro | Recessive | WES | 0,33 | 8 | Male | White (European) | yes | 15 |
|  | *PDE10A* | #616921 - Dyskinesia, limb and orofacial, infantile-onset | c.346G>C | p.Ala116Pro | Recessive | WES | 0,33 | 7 | Male | White (European) | yes | 15 |
|  | *PTPS/*  *PTS* | #261640 - Hyperphenylalaninemia, BH4-deficient, 6-pyruvoyl-tetrahydropterin synthase deficiency | c.243_243+1dup | Predicted splicing effect - donor site lost | Recessive | Single gene testing | 0,33 | 0,6 | Female | White (European) | No | No |
|  | *PTPS/*  *PTS* | #261640 - Hyperphenylalaninemia, BH4-deficient, 6-pyruvoyl-tetrahydropterin synthase deficiency | c.25C>T/  c.260C>T | p.Arg9Cys/p.Pro87Leu | Recessive | Single gene testing | 16 | 32 | Male | White (European) | No | 16 |
|  | *PTPS/*  *PTS* | #261640 - Hyperphenylalaninemia, BH4-deficient, 6-pyruvoyl-tetrahydropterin synthase deficiency | c.139A>G, c.338A>G | p.Asn47Asp, p.Tyr113Cys | Recessive | Single gene testing | 0,66 | 0,75 | Female | White (European) | No | No |
|  | *PTPS/*  *PTS* | #261640 - Hyperphenylalaninemia, BH4-deficient, 6-pyruvoyl-tetrahydropterin synthase deficiency | c.53T>C/  c.139A>G | p.Ile18Thr/  p.Asn47Asp | Recessive | Single gene testing | 0,08 | 6 | Male | White (European) | No | 16 |
|  | *SGCE* | #159900 - Myoclonus-dystonia syndrome (DYT11) | c.662G>A | p.Gly221Asp | Dominant inherited (father) | Single gene testing | 3 | 6 | Female | White (European) | No | 17 |
|  | *SGCE* | #159900 - Myoclonus-dystonia syndrome (DYT11) | c.662G>A | p.Gly221Asp | Dominant inherited (father) | Single gene testing | 7 | 68 | Female | White (European) | No | 17 |
|  | *SGCE* | #159900 - Myoclonus-dystonia syndrome (DYT11) | c.1037+5G>A | Predicted splicing effect - donor site lost | Dominant inherited (father) | Single gene testing | 11 | 19 | Female | White (European) | No | 17 |
|  | *SGCE* | #159900 - Myoclonus-dystonia syndrome (DYT11) | c.1037+5G>A | Predicted splicing effect - donor site lost | Dominant inherited (father) | Single gene testing | 4,5 | 18 | Female | White (European) | No | 17 |
|  | *SGCE* | #159900 - Myoclonus-dystonia syndrome (DYT11) | c.1037+5G>A | Predicted splicing effect - donor site lost | Dominant inherited (father) | Single gene testing | 10 | 63 | Male | White (European) | No | 17 |
|  | *SGCE* | #159900 - Myoclonus-dystonia syndrome (DYT11) | c.289C>T | p.Arg97* | Dominant inherited (father) | Single gene testing | 8,5 | 30 | Male | White (European) | No | 17 |
|  | *SGCE* | #159900 - Myoclonus-dystonia syndrome (DYT11) | c.942C>G | p.Tyr314* | Dominant inherited (father) | Single gene testing | 2 | 15 | Male | White (European) | No | 17 |
|  | *SGCE* | #159900 - Myoclonus-dystonia syndrome (DYT11) | c.942C>G | p.Tyr314* | Dominant inherited (father) | Single gene testing | 4,5 | 61 | Female | White (European) | No | 17 |
|  | *SGCE* | #159900 - Myoclonus-dystonia syndrome (DYT11) | Del chr7:92180000-94250000, including PEG10, SGCE, CASD1, COL1A2, BET1, GNG11, TFP12, GNG1, CALCR, HCTR-6, KIAA 1861, CCDC132, HEPACAM2, SAMD9, SAMD9L | Whole gene deletion | Dominant inherited (father) | arrayCGH | 2,5 | 8 | Female | White (European) | No | 17 |
|  | *SGCE* | #159900 - Myoclonus-dystonia syndrome (DYT11) | Del chr7:92180000-94250000, including PEG10, SGCE, CASD1, COL1A2, BET1, GNG11, TFP12, GNG1, CALCR, HCTR-6, KIAA 1861, CCDC132, HEPACAM2, SAMD9, SAMD9L | Whole gene deletion | Dominant inherited (father) | arrayCGH | 2 | 4 | Female | White (European) | No | 17 |
|  | *SGCE* | #159900 - Myoclonus-dystonia syndrome (DYT11) | Del chr7:92330000-94670000, including PPP1R9A, PEG10, SGCE, CASD1, COL1A2, BET1, GNG11, TFP12, GNG1, CALCR, HCTR-6, KIAA 1861, CCDC132, HEPACAM2, SAMD9, SAMD9L | Whole gene deletion | Dominant inherited (father) | arrayCGH | 4 | 9 | Male | White (European) | No | 17 |
|  | *SGCE* | #159900 - Myoclonus-dystonia syndrome (DYT11) | Del chr7:92330000-94670000, including PPP1R9A, PEG10, SGCE, CASD1, COL1A2, BET1, GNG11, TFP12, GNG1, CALCR, HCTR-6, KIAA 1861, CCDC132, HEPACAM2, SAMD9, SAMD9L | Whole gene deletion | Dominant inherited (father) | arrayCGH | 2 | 3 | Female | White (European) | No | 17 |
|  | *SGCE* | #159900 - Myoclonus-dystonia syndrome (DYT11) | c.771_772del | p.Cys258* | Dominant inherited (father) | Single gene testing | 2,5 | 50 | Female | White (European) | No | 17 |
|  | *SGCE* | #159900 - Myoclonus-dystonia syndrome (DYT11) | c.942C>G | p.Tyr314* | Dominant (parental status unknown) | Single gene testing | 1,5 | 44 | Female | White (European) | No | 17 |
|  | *SGCE* | #159900 - Myoclonus-dystonia syndrome (DYT11) | c.289C>T | p.Arg97* | Dominant inherited (father) | Single gene testing | 2 | 28 | Male | White (European) | No | 17 |
|  | *SGCE* | #159900 - Myoclonus-dystonia syndrome (DYT11) | c.630_658del | p.Val211Glyfs*20 | Dominant (parental status unknown) | Single gene testing | 10 | 54 | Male | White (European) | No | 17 |
|  | *SGCE* | #159900 - Myoclonus-dystonia syndrome (DYT11) | c.289C>T | p.Arg97* | Dominant inherited (father) | Single gene testing | 1,5 | 47 | Female | White (European) | No | 17 |
|  | *SGCE* | #159900 - Myoclonus-dystonia syndrome (DYT11) | c.109+5G>C | Predicted splicing effect - donor site likely lost | Dominant inherited (father) | Single gene testing | 2 | 10 | Male | White (European) | No | 17 |
|  | *SGCE* | #159900 - Myoclonus-dystonia syndrome (DYT11) | Del Chr7:94248070-94248268 | Whole exon 5 deletion | Dominant (parental status unknown) | Single gene testing | 3 | 5 | Female | White (European) | No | 17 |
|  | *SGCE* | #159900 - Myoclonus-dystonia syndrome (DYT11) | c.348delG | p.Ser117Profs*15 | Dominant inherited (father) | Single gene testing | 1,33 | 6,91 | Male | White (European) | No | No |
|  | *SGCE* | #159900 - Myoclonus-dystonia syndrome (DYT11) | c.109+3dupT | Predicted splicing effect - donor site likely lost | Dominant inherited (father) | Single gene testing | 1,7 | 4 | Male | White (European) | No | No |
| 85 | *SGCE* | #159900 - Myoclonus-dystonia syndrome (DYT11) | c.734_737delAATT | p.Gln245Argfs*10 | Dominant de novo | Single gene testing | 2 | 12 | Female | White (European) | No | No |
| 86 | *SGCE* | #159900 - Myoclonus-dystonia syndrome (DYT11) | c.308delA | p.Tyr103Leufs*29 | Dominant inherited (father) | Single gene testing | 1,3 | 3 | Male | White (European) | No | No |
| 87 | *SGCE* | #159900 - Myoclonus-dystonia syndrome (DYT11) | c.289C>T | p.Arg97Ter | Dominant de novo | Single gene testing | 1,5 | 17 | Male | White (European) | No | No |
| 88 | *SGCE* | #159900 - Myoclonus-dystonia syndrome (DYT11) | c.703delT | p.Cys235Valfs*12 | Dominant inherited (father) | Single gene testing | 2 | 2,5 | Female | White (European) | No | No |
|  | *SGCE* | #159900 - Myoclonus-dystonia syndrome (DYT11) | c.709C>T | p.Arg237* | Dominant inherited (father) | Single gene testing | 3 | 7 | Male | White (European) | No | No |
|  | *SGCE* | #159900 - Myoclonus-dystonia syndrome (DYT11) | c.911delC | p.Pro304Leufs*5 | Dominant inherited (father) | Single gene testing | 1 | 4 | Male | White (European) | No | No |
|  | *SGCE* | #159900 - Myoclonus-dystonia syndrome (DYT11) | c.289C>T | p.Arg97* | Dominant inherited (father) | Single gene testing | 11 | 62 | Male | White (European) | No | 17 |
|  | *SGCE* | #159900 - Myoclonus-dystonia syndrome (DYT11) | c.289C>T | p.Arg97* | Dominant inherited (father) | Single gene testing | 3 | 31 | Female | White (European) | No | 17 |
|  | *SGCE* | #159900 - Myoclonus-dystonia syndrome (DYT11) | c.289C>T | p.Arg97* | Dominant inherited (father) | Single gene testing | 1,5 | 22 | Female | White (European) | No | 17 |
|  | *SLC2A1* | #606777 - GLUT1 deficiency syndrome | c.874T>C | p.Tyr292Hys | Dominant de novo | Single gene testing | 1,5 | 6 | Female | White (European) | No | No |
|  | *SLC2A1* | #606777 - GLUT1 deficiency syndrome | c.1214_1215insAGGCTTCTCCAACTGGACCTCAAATTTCATTGTGGGCATGTGCTTCCAGTATGTGGAGGTGAGAACCCCCACTGTCTCTATA | p.Val406Glyfs*76 | Dominant inherited | Single gene testing | 2 | 4 | Female | White (European) | No | No |
|  | *SLC2A1* | #606777 - GLUT1 deficiency syndrome | c.277C>T | p.Arg93Trp | Dominant (parenal status unknown) | Single gene testing | 4 | 14 | Female | White (European) | No | No |
| 104 | *SLC2A1* | #606777 - GLUT1 deficiency syndrome | c.876_878dupTTA | p.Tyr293dup | Dominant de novo | Single gene testing | 5 | 7 | Female | White (European) | No | 18 |
|  | *SLC6A3* | #613135 - Infantile parkinsonism-dystonia_Dopamine Transporter Deficiency Syndrome | c.1184C>T Homozygous | p.Pro395Leu | Recessive | Single gene testing | 0,4 | 10 | Female | White (European) | Yes - second cousins | 19 |
|  | *SLC6A3* | #613135 - Infantile parkinsonism-dystonia_Dopamine Transporter Deficiency Syndrome | c.1103T>A Homozygous | p.Leu368Gln | Recessive | Single gene testing | 0,3 | 4 | Male | Asian (Pakistani) | Yes - first cousins | 19 |
|  | *SLC6A3* | #613135 - Infantile parkinsonism-dystonia_Dopamine Transporter Deficiency Syndrome | c.1156+5delG Homozygous | Predicted splicing effect - donor site likely lost - possible skip of exon 8 | Recessive | Single gene testing | 0,3 | 8 | Female | White (European) | No | 20 |
|  | *SLC6A3* | #613135 - Infantile parkinsonism-dystonia_Dopamine Transporter Deficiency Syndrome | 399delG Homozygous | p.Ile134Serfs*5 | Recessive | Single gene testing | 0,12 | 0,6 | Female | White (European) | yes | 20 |
|  | *SLC6A3* | #613135 - Infantile parkinsonism-dystonia_Dopamine Transporter Deficiency Syndrome | 1499_1767del Homozygous | p.Gly500Glufs*13 | Recessive | Single gene testing | 0,04 | 2 | Female | White (European) | No | 20 |
|  | *SLC6A3* | #613135 - Infantile parkinsonism-dystonia_Dopamine Transporter Deficiency Syndrome | 671T>C Homozygous | p.Leu224Pro | Recessive | Single gene testing | 0,6 | 1,9 | Female | White (European) | yes | 20 |
|  | *SLC6A3* | #613135 - Infantile parkinsonism-dystonia_Dopamine Transporter Deficiency Syndrome | c.941C>T Homozygous | p.Ala314Val | Recessive | WES | 11 | 16 | Male | Asian (Pakistani) | Yes - first cousins | 21 |
|  | *SLC6A3* | #613135 - Infantile parkinsonism-dystonia_Dopamine Transporter Deficiency Syndrome | c.941C>T Homozygous | p.Ala314Val | Recessive | WES | 11 | 26 | Male | Asian (Pakistani) | Yes - first cousins | 21 |
|  | *SLC6A3* | #613135 - Infantile parkinsonism-dystonia_Dopamine Transporter Deficiency Syndrome | c.941C>T Homozygous | p.Ala314Val | Recessive | WES | 11 | 28 | Male | Asian (Pakistani) | Yes - first cousins | 21 |
|  | *SLC6A3* | #613135 - Infantile parkinsonism-dystonia_Dopamine Transporter Deficiency Syndrome | c.287-5_287-2delinsAAC | Predicted splicing effect - acceptor site lost - very likely skip of exon 3 | Recessive | WES | 0,25 | 4 | Male | White (European) | No | 21 |
|  | *SPR* | #612716 - Dopa responsive dystonia_ Sepiapterin Reductase Deficiency | c.596-2A>G Homozygous | Predicted splicing effect - acceptor site lost | Recessive | Single gene testing | 0,25 | 13 | Male | White (European) | Unknown | 22 |
|  | *SPR* | #612716 - Dopa responsive dystonia_ Sepiapterin Reductase Deficiency | c.448A.G and c.751A.T | p.Arg150Gly /p.Lys251* | Recessive | Single gene testing | 0 | 0,5 | Female | White (European) | No | 23 |
|  | *SPR* | #612716 - Dopa responsive dystonia_ Sepiapterin Reductase Deficiency | c.305-2A>G Homozygous | Predicted splicing effect - acceptor site lost - very likely skip of exon 2 | Recessive | Single gene testing | 5 | 13 | Male | Asian (Indian) | Yes | No |
|  | *SPR* | #612716 - Dopa responsive dystonia_ Sepiapterin Reductase Deficiency | c.305-2A>G Homozygous | Predicted splicing effect - acceptor site lost - very likely skip of exon 2 | Recessive | Single gene testing | 1 | 15 | Male | Asian (Indian) | Yes | No |
|  | *TH* | #605407 - Dopa responsive dystonia autosomal recessive_Tyrosine hydroxylase deficiency | c.983G>A/  c.1211C>T | p.Arg328Gln/  p.Thr404Met | Recessive | Single gene testing | 0,25 | 3,5 | Male | White (European) | No | No |
|  | *TH* | #605407 - Dopa responsive dystonia autosomal recessive_Tyrosine hydroxylase deficiency | c.707T>C homozygous | p.Leu236Pro | Recessive | Single gene testing | 0,41 | 0,6 | Female | White (European) | No | 24 |
|  | *TH* | #605407 - Dopa responsive dystonia autosomal recessive_Tyrosine hydroxylase deficiency | c.707T>C homozygous | p.Leu236Pro | Recessive | Single gene testing | 0,41 | 0,5 | Female | White (European) | No | 24 |
| 113 | *TH* | #605407 - Dopa responsive dystonia autosomal recessive_Tyrosine hydroxylase deficiency | c.707T>C homozygous | p.Leu236Pro | Recessive | Single gene testing | 0,2 | 2,2 | Female | White (European) | No | 24 |
|  | *TH* | #605407 - Dopa responsive dystonia autosomal recessive_Tyrosine hydroxylase deficiency | c.707T>C homozygous | p.Leu236Pro | Recessive | Single gene testing | 0,25 | 0,5 | Female | White (European) | No | 24 |
|  | *TH* | #605407 - Dopa responsive dystonia autosomal recessive_Tyrosine hydroxylase deficiency | c.1240G>A/  c.1529T>A | p.Gly414Arg/p.Leu510Gln | Recessive | Single gene testing | 1 | 13 | Male | White (European) | No | 25 |
|  | *TH* | #605407 - Dopa responsive dystonia autosomal recessive_Tyrosine hydroxylase deficiency | c.1076G>T homozygous | p.Cys359Phe | Recessive | Single gene testing | 0,33 | 6 | Male | White (European) | Yes | 26 |

*ADCY5* (NM_183357.2), *ATP1A3* (NM_152296.4), *DDC* (NM_001082971.1), *DHPR/QDPR (*NM_000320.2), *FOXG1* (NM_005249.4), *GCH1* (NM_001024024.1), *GNAO1* (NM_020988.2), *KMT2B* (NM_014727.2), *MICU1* (NM_006077.3), *NKX2-1* (NM_001079668.2), *PDE10A* (NM_001130690.2), *PTPS/PTS* (NM_000317.2), *SGCE* (NM_003919.2), *SLC2A1* (NM_006516.2), *SLC6A3* (NM_001044.4), *SPR* (NM_003124.4), *TH* (NM_199292.2); aCGH (array comparative genomic hybridisation), WGS (whole genomes sequencing), WES (whole exome sequencing)

**Supplementary Table 2:** Functional impact, associated clinical features and long-term outcome in patients with complex genetic HMD.

|  | Impairment on DLAs | | | | Other neurological and systemic clinical features | Developmental delay  Intellectual disability | Disease course and outcome |
| --- | --- | --- | --- | --- | --- | --- | --- |
|  | Speech | Fine motor skills  (W, F, H, D) | Gross motor skills (gait) | Eating/  Swallowing |  |  |  |
| *ADCY5*  n=4 | Dysarthria (2)  Paroxysmal dysarthria (1) | Dependent (1) | Impaired gait (2)  Assisted gait (1) | Dysphagia (1) | Hypotonia (1) | Mod DD (1) | Static (2)  Progressive in early childhood (2) |
| *ATP1A3*  n=6 | Dysarthria (5) | Writing difficulties (6)  Dependent (3) | Impaired gait (4)  Cannot walk (1) | Dysphagia (2)  [during attacks] | Hypotonia (3)  Pyramidal signs (2)  Seizures (2) | Mild-Mod (3) –severe (2) DD/ID | Static (4)  Progressive (2) |
| *FOXG1*  n=17 | Anarthria (11)  Dysarthria (6) | Dependent (13) | Cannot walk (11)  Assisted gait (2) | Dysphagia (12) | Seizures (16)  Microcephaly (12)  Hypotonia (4) | Mild-Mod (4)-Severe (13) DD/ID | Static (4)  Progressive (6) |
| *GNAO1*  n=7 | Anarthria (3)  Dysarthria (4) | Dependent (7) | Cannot walk (5)  Impaired gait (1)  Assisted gait (1) | Dysphagia (4) | Severe hypotonia (6)  Microcephaly (2)  Pyramidal signs (2)  Seizures (4) | Mild-Mod (2)-Severe (7) DD/ID | Progressive (6)  Static (1)  Severe exacerbations of HMD (4) |
| *KMT2B*  n=16 | Dysphonia/dysarthria (15)  Anarthria (1) | Dependent (16) | Impaired/assisted gait (12)  Can walk after DBS (4) | Dysphagia (10) | Short stature (2)  Pyramidal signs (1)  Cutis aplasia (1) Hypertrichosis (1)  Retinal dystrophy (1)  Ectodermal dysplasia (1)  Fifth-finger clinodactyly (1)  Cleft palate (1)  Strabismus (1)  Phimosis (1)  Episodic vomiting (1)  Ichtyotic skin lesion (1) | Mild ID (8)  Moderate ID (1)  Normal development in all cases | Progressive dystonia (14)  Static (2)  Status dystonicus (2) |
| *MICU1*  n=2 | Dysarthria (1) | Dependent (1)  Writing difficulties (1) | Impaired gait (2) | None | Mild proximal weakness (2)  Lax joints-pes planus (2)  Severe myopia (2)  Short stature (1)  Microcephaly (1) | Mild DD/ ID (2) | Static (2) |
| *NKX2*.1  n=8 | None | Writing difficulties (7) Dependent (3) | impaired gait (8) | None | Hypotonia in infancy (7)  Hypothyroidism (3)  Anxiety, depression (2)  ADHD (1)  Respiratory disease (1)  Hypodontia (1)  Macrocephaly (1) | Mild motor DD (5) | Static (8) |
| *PDE10A*  n=2 | Severe dysarthria (2) | Dependent (2) | Assisted gait (1)  Cannot walk (1) | Dysphagia (1) | Hypotonia (2)  IDDM (1)  Seizures (1) | Mild-mod DD (2) | Static (2) |
| *SGCE*  n=30 | Dysarthria (3) | Writing difficulties (26), Problems using cutlery (22) Hygiene and dressing (7) | Impaired gait (11) | None | OCD (17)  Anxiety disorder (14)  Depression (11)  Phobia (11)  Panic disorder (8)  Alcohol dependence (7)  Psychosis (1)  Short stature (5)  Seizures (1) | Mild-Mod DD (5) | Static (25)  Progressive (3)  Improvement (1) |
| *SLC2A1*  n=4 | Language delay, dysarthria (1) | Writing difficulties (3)  Dependent (1) | Impaired gait (2) |  | Seizures (3)  Pyramidal signs (3)  Microcephaly (2)  Ataxia (2)  Strabismus (1)  ADHD (1) | Mild-Mod DD (4) | Static (4) |
| *Neurotransmitter defects* | | | | | | | |
| *DDC*  n=9 | Language delay (9)  Dysarthria (2)  NA (2) | Writing difficulties (2) Dependent (3)  NA (4) | Cannot walk (6)  Impaired gait (2)  NA (2) | Dysphagia (6) | Hypotonia (7)  Autonomic features (7)  Chest infections (3)  GOR (3)  Pyramidal signs (3)  Failure to thrive (2)  Microcephaly (2)  Brachycephaly (2) Myaesthenia-like (2) Strabismus (1)  Orthostatic hypotension (1) Irregular menstruation (1) | Mild-Mod (2)-Severe DD (7)  Borderline ID (2) | Improvement (9) |
| *DHPR*  n=4 | Dysphonia (1)  NA (3) | NA (3) | NA (3) | Dysphagia (1) | Seizures (2) | Mild-Mod (3) - Severe (1) DD | Improvement (3)  Progressive epilepsy (1) |
| *GCH1*  n=6 | Dysarthria (2) | Writing difficulties (3) Dependent (2) | Impaired gait (6) |  | Pyramidal signs (1) Kyphoscoliosis (1)  Depression/anxiety (2)  Seizures (1) |  | Improvement (6) |
| *PTPS*  n=4 | Language delay (1)  Palilalia, dysarthria (1)  NA (2) | Writing difficulties (2)  NA (2) | Impaired gait (1)  NA (2) | Dysphagia (1) | Microcephaly (1)  Strabismus (1) | Mild-Mod DD (3) | Improvement (4) |
| *SLC6A3*  n=10 | Anarthria (6)  Speech regression (2)  NA (2) | Dependent (6)  NA (2) | Cannot walk (6)  Impaired gait (1)  NA (2) | Dysphagia (7) | Axial hypotonia (7)  Bulbar dysfunction (7)  Failure to thrive (7) Pyramidal signs (3) | Severe DD (7) | Progressive (10)  Status dystonicus (1) |
| *SPR*  n=4 | Language delay (2)  Scanning speech (2)  NA (1) | Dependent (2)  NA (1) | NA(1) | Dysphagia (2) | Microcephaly (1) Brachycephaly (1)  Pyramidal signs (1)  Autism (1) | DD before levodopa (3) | Improvement (4) |
| *TH*  n=7 | Dysarthria (5)  Language delay (3)  NA (3) | Writing difficulties (7) Dependent (2)  NA (3) | Impaired gait (2)  Assisted gait (1)  Cannot walk (1)  NA (3) | Dysphagia (2) | Autonomic features (7)  Psychiatric features (3)  Failure to thrive (1)  Microcephaly (1)  Pyramidal signs (1)  Oculomotor apraxia (1) Seizures (1) | DD before levodopa (7) Borderline ID (1) | Improvement (7) |

Daily living activities (DLAs) requiring fine motor skills: writing (W), using cuterly for feeding (F), hygiene (H) and dressing (D), independent or dependent (if any help was needed to perform these activities). Gait: impaired gait (independent but slow gait, frequent falls or imbalance), Assisted gait (requires an assistance device for safe walking), cannot walk (cannot walk at all or only with another person’s assistance), NA: Not Applicable (≤ 2 years) DD Developmental delay; GOR: gastro-oesophageal reflux; ID intellectual disability; IDDM: insulin dependent diabetes mellitus, Mod - moderate

**Supplementary Table 3:** Impact of complex genetic HMD on daily living activities

| **Genes** | **Speech** | **Fine motor skills**  **(F, H, D)** | **Gross motor skills (Gait)** | **Eating and swallowing** |
| --- | --- | --- | --- | --- |
| *ADCY5* |  |  |  |  |
| *ATP1A3* |  |  |  |  |
| *FOXG1* |  |  |  |  |
| *GNAO1* |  |  |  |  |
| *KMT2B* |  |  |  |  |
| *MICU1* |  |  |  |  |
| *NKX2.1* |  |  |  |  |
| *PDE10A* |  |  |  |  |
| *SGCE* |  |  |  |  |
| *SLC2A1* |  |  |  |  |
| **Neurotransmitter defects** | | | | |
| *DDC* |  |  |  |  |
| *DHPR* |  |  |  |  |
| *GCH* |  |  |  |  |
| *PTPS* |  |  |  |  |
| *SLC6A3* |  |  |  |  |
| *SPR* |  |  |  |  |
| *TH* |  |  |  |  |

**Footnote Supplementary Table 3.** Percentage of patients with functional impact of the HMD on their daily living activities (DLA). Patients were scored according to the age at assessment as follows: for speech (patients older than 3 years, n=121); for daily living activities requiring fine motor tasks (i.e. using cutlery for feeding, hygiene and dressing) (patients older than 5 years, n=103); for gait impairment (patients older than 2 years, n=128; for eating and swallowing (all patients, n=140).

Colour shading indicates the prevalence (%) of impacted DLAs as follows:

| 0-24% | 25-49% | 50-74% | 75-100% |
| --- | --- | --- | --- |

**Supplementary Table 4:** Complex genetic HMD presenting with paroxysmal events or exacerbations, and associated triggers.

| **Genes**  **(Triggers)** | | **Oculogyric Crises** | **Dystonic Attacks** | **Status Dystonicus/**  **Hyperkinetic status** | | **Exercise-induced dystonia** | **Plegic attacks** | **Paroxysmal ataxia** | | **Paroxysmal choreoathetosis** | **Myoclonus** |
| --- | --- | --- | --- | --- | --- | --- | --- | --- | --- | --- | --- |
| *ADCY5*  *Drowsiness and Sleep/Emotion/Fatigue/Illness* | |  |  |  | |  |  |  | |  |  |
| *ATP1A3*  *Fatigue/Emotion/Temperature* | |  |  |  | |  |  |  | |  |  |
| *FOXG1* | |  |  |  | |  |  |  | |  |  |
| *GNAO1*  *Fever/illness/Emotion/Temperature/Movements* | |  |  |  | |  |  |  | |  |  |
| *KMT2B*  *Exercise/Illness* | |  |  |  | |  |  |  | |  |  |
| *MICU1* | |  |  |  | |  |  |  | |  |  |
| *NKX2.1* | |  |  |  | |  |  |  | |  |  |
| *PDE10A* | |  |  |  | |  |  |  | |  |  |
| *SGCE*  *Emotion* | |  |  |  | |  |  |  | |  |  |
| *SLC2A1*  *Exercise/Fatigue/Fasting/Illness* | |  |  |  | |  |  |  | |  |  |
| **Neurotransmitter defects** |  | | | |  | | | |  | |  |
| *DDC*  *Diurnal/Fatigue/Fasting/Illness/Emotion* | |  |  |  | |  |  |  | |  |  |
| *DHPR*  *Diurnal* | |  |  |  | |  |  |  | |  |  |
| *GCH1*  *Diurnal/Exercise/Emotion/Temperature* | |  |  |  | |  |  |  | |  |  |
| *PTPS*  *Diurnal* | |  |  |  | |  |  |  | |  |  |
| *SLC6A3* | |  |  |  | |  |  |  | |  |  |
| *SPR*  *Diurnal/Fatigue/Illness/Emotion* | |  |  |  | |  |  |  | |  |  |
| *TH*  *Diurnal/Fatigue* | |  |  |  | |  |  |  | |  |  |

Columns represent phenotype features and raws represent causal factors. Boxes in grey indicate the existence of an association between causal gene and a certain phenotype.
